# Assessing biological network dynamics: comparing numerical simulations with analytical decomposition of parameter space

**DOI:** 10.1038/s41540-023-00289-2

**Published:** 2023-07-03

**Authors:** Kishore Hari, William Duncan, Mohammed Adil Ibrahim, Mohit Kumar Jolly, Breschine Cummins, Tomas Gedeon

**Affiliations:** 1 Centre for BioSystems Science and Engineering, Indian Institute of Science, Bangalore, 560012 India; 2 Department of Mathematical Sciences, Montana State University, Bozeman, MT 59717 USA; 3 Department of Chemical Engineering, Indian Institute of Technology Hyderabad, Hyderabad, 502285 India

**Keywords:** Biochemical networks, Dynamical systems

## Abstract

Mathematical modeling of the emergent dynamics of gene regulatory networks (GRN) faces a double challenge of (a) dependence of model dynamics on parameters, and (b) lack of reliable experimentally determined parameters. In this paper we compare two complementary approaches for describing GRN dynamics across unknown parameters: (1) parameter sampling and resulting ensemble statistics used by RACIPE (RAndom CIrcuit PErturbation), and (2) use of rigorous analysis of combinatorial approximation of the ODE models by DSGRN (Dynamic Signatures Generated by Regulatory Networks). We find a very good agreement between RACIPE simulation and DSGRN predictions for four different 2- and 3-node networks typically observed in cellular decision making. This observation is remarkable since the DSGRN approach assumes that the Hill coefficients of the models are very high while RACIPE assumes the values in the range 1-6. Thus DSGRN parameter domains, explicitly defined by inequalities between systems parameters, are highly predictive of ODE model dynamics within a biologically reasonable range of parameters.

## Introduction

As the sophistication and scope of experimental methods in molecular and cell biology continues to grow, there is an increased need to use this data to synthesize a coherent understanding at the systems level. Experimental methods are successful if they isolate and study a particular feature of a complex system in isolation. The need to understand function of complex systems “as a whole" by combining partial insights is a goal of systems biology^1^. One of the principal demonstrations of the fact that such a unified insight has been achieved is to construct a mathematical model.

In this paper, we discuss dynamical models that are built to represent the behavior of networks. Networks are often constructed from experimental data by postulating pairwise interactions of chemical species, e.g genes, their products, proteins and other molecules. The methodologies for discovery of such connections have varied levels of reliability, but none of them can measure the interaction between multiple effectors, or the range of behaviors of the network under varying conditions. Mathematical models are asked to integrate the local pairwise interactions and make predictions about the network behavior in conditions that are not directly experimentally accessible^2^.

The principal challenge for model construction and validation is parameterization. The network structure constrains potential dynamics but does not uniquely determine it. In fact, the behavior of the network in different conditions, perhaps embedded in different individual cells, may be different precisely because the underlying kinetic parameters have changed. While there are several methods to determine the structure of the network, it is very difficult to measure parameters, especially because they may depend on precise experimental conditions. Because of this, even comparing a model prediction to an experiment is challenging; if the parameters for the experiment do not agree with the parameters of the model when simulated, a correct model may give a disappointing fit. On the other hand, having a good fit does not guarantee that the model is generalizable beyond the conditions that have been fit. Therefore, validation of a model should require that the description of the behavior of the model is provided not only for particular set of parameters, or for a particular initial condition, but includes a broad range of potential dynamics across parameters and initial conditions.

In this paper we compare two different approaches to describing a range of potential dynamics of a network. RACIPE^3^ relies on random but judicious sampling of parameters and initial conditions with an ODE (Ordinary Differential Equation) based simulation that describes the interactions in the form of Hill functions, while DSGRN^4–7^ uses combinatorial computations to analyze all multi-level Boolean models compatible with the network dynamics. DSGRN embeds^8^ discrete Boolean models into a continuous framework of switching systems^9–16^, which then permits the use of ideas from bifurcation theory to understand changes in dynamics as a function of parameters. This close relationship between Boolean and ODE descriptions leads to rigorous mathematical results that link dynamics described by DSGRN and that of smooth ODE systems with sufficiently steep nonlinearities^17,18^. There are also explicit results for the size of allowed perturbations of equilibria predicted by DSGRN to systems with ramp nonlinearities^19^. DSGRN has been used successfully to describe complex dynamics of networks ranging from cell cycle^20^ to EMT network^21^. RACIPE has been used to describe the dynamics of networks of varying sizes and biological contexts to understand the role of network topology in leading to various emergent phenotypes^22–24^. However, the role of parameters in the emergence of various phenotypes remains to be understood.

There is a long tradition of comparing the dynamics of continuous-time ODE models and discrete time Boolean models or more general multilevel discrete models. The pioneering study^25^ showed remarkable robustness of the segment polarity gene network in *Drosophila melanogaster* modelled by a large system of ODEs. This was followed by a study of the Boolean model^26^ of the same system that was also able to reproduce both WT and several mutant expression patterns, supporting the notion that the source of robustness is in network topology. A more recent effort uses the HillCubes approach^27^ to approximate Boolean functions by ODE models. The resulting software Odefy^28^ takes a Boolean model and produces a corresponding ODE model. These models show good correspondence on the T-cell receptor signaling model^27^; the same construction was used in ref. ^29^ to observe correspondence in dynamics between discrete and continuous models of the human cell cycle.

In this paper we compare RACIPE and DSGRN approaches to study dynamics of gene regulatory networks (GRNs). In Section “Analysing RACIPE data to identify parameter attractor-repertoire bound-aries” we use RACIPE to generate parameter samples and simulate dynamics of three two-node networks: Toggle Switch (TS), Double Activation (DA) and Negative Feedback loop (NF). We attempt to find parameters of the network that would predict behaviors like monostability vs. bistability. We do not observe any clear associations between individual parameters and GRN dynamic behavior. We hypothesise that the reason is that such behaviors depend on combinations of parameters, rather than on a single parameter. Since a combinatorial explosion prevents us from testing predictions based on all possible combinations of several parameters, we turn to DSGRN methodology. There is a direct translation between RACIPE parameters and DSGRN parameters, with the exception of the Hill coefficient *n* that is a parameter in RACIPE, but is missing in the DSGRN switching ODE model since this model corresponds to the RACIPE model in the limit *n* → *∞*. However, DSGRN provides explicit decomposition of the parameter space (Fig. 4) into domains that have invariant dynamical behavior, which is directly computable without use of ODE simulations.

We are therefore able to compare RACIPE simulations to DSGRN predictions by locating the RACIPE parameter within a particular DSGRN parameter domain. We find a very tight fit for when the RACIPE samples Hill coefficients from range 10–100. Perhaps surprisingly, we also find that this fit does not deteriorate much when we sample Hill coefficients from range 1–10. Since this lower range is biologically more plausible, this suggests that the DSGRN parameter domain decomposition predicts dynamics for the biological range of Hill coefficients *n*.

We then test the close match between RACIPE simulations and DSGRN predictions on a three-node network Toggle Triad (TT). We find the same above mentioned good agreement.

Finally, we test an ensemble agreement between these two approaches. To do this we ask whether the frequency of dynamical behavior pooled across all parameter samples of RACIPE matches the frequency of dynamical behavior pooled across all parameter nodes of DSGRN. We find disagreement that persists for all values of *n*. We are able to fully explain this disagreement as caused by a non-uniform sampling of individual DSGRN parameter domains by RACIPE. In particular, for the examined two-node networks, the DSGRN parameter node that predicts bistability is sampled much more often than the nodes that predict monostability. When corrected for the nonuniform sampling, the agreement between DSGRN predictions and RACIPE simulations is restored.

By integrating the ideas from RACIPE and DSGRN, our results provide a way to deepen our understanding of the boundaries in the parameter space between distinct dynamical behaviors.

## Results

### Analysing RACIPE data to identify parameter attractor-repertoire boundaries

Using RACIPE, a parameter-agnostic approach that estimates the steady states of gene regulatory networks over a large parameter space, we aim to understand the regions of parameters that lead to a particular dynamical behavior of the network. Each parameter set specifies a system of differential equations whose dynamics in the attractor-repertoire space exhibits a long term behavior like steady states, periodic oscillations and multistability. In order for these behaviors to be observable, they must be stable, i.e., they must attract nearby initial conditions. We will use the word “attractor-repertoire" to denote different types of stable behavior of a system. Therefore the goal is to describe for each network a collection of attainable attractor-repertoires together with description of parameter domains that parameterize systems with that attractor-repertoire (See Methods).

**Fig. 1:**
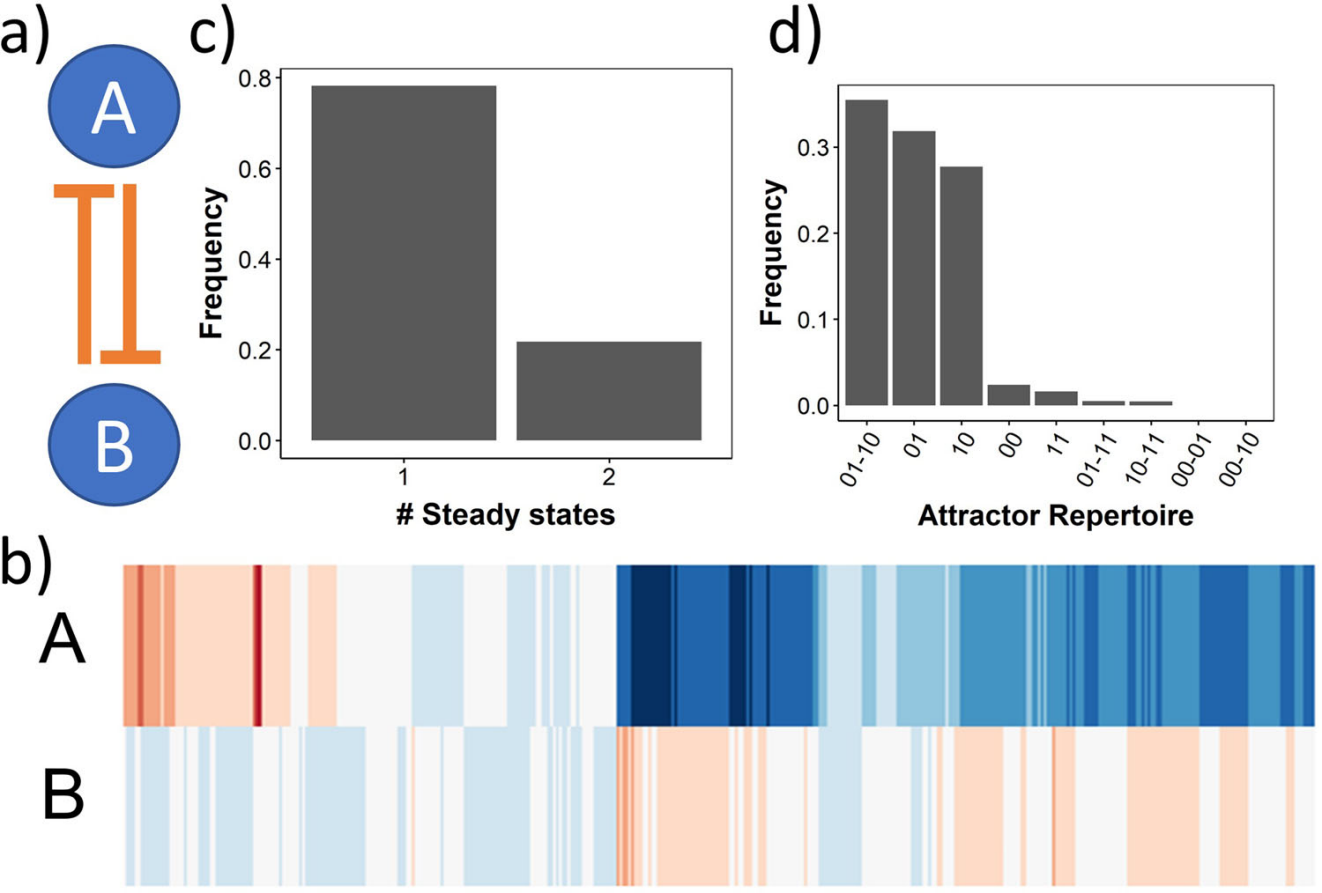
Output of RACIPE for Toggle Switch (TS) network. **a** TS network structure. **b** Heatmap representing all solutions from all parameter sets obtained via RACIPE. Red color indicates high, white indicates moderate and blue indicates lower expression levels of the node variable labelled to the left. **c** Frequency of monostable (x-axis label 1) and bistable (x-axis label 2) parameter sets sampled by RACIPE. **d** Frequency distribution of individual attractor-repertoires obtained from RACIPE for TS.

We start by analysing a simple gene regulatory network called the Toggle Switch (TS): a network with two nodes and two edges, or equivalently “links”, such that each node inhibits the production of the other (Fig. 1a). In the context of GRNs, nodes can be transcription factors or RNA molecules. We simulated TS using RACIPE, obtaining a map between parameter sets and the corresponding steady states (Fig. 1b). We discretized these steady-state values for ease of analysis, so that each steady state belongs to one of the four categories: high-high (11), high-low (10), low-high (01) and low-low (00). High and low levels of RACIPE steady states are defined based on whether the steady state level is higher (1) or lower (0) than the ensemble mean, where the ensemble is identified by the collection of parameter sets sampled by RACIPE. Simulating TS using RACIPE gives us two types of behavioral information: a) the number of steady states emergent from each parameter set (Fig. 1c, b) the category of the steady state(s) given by each parameter set (Fig. 1d). The dominant dynamical behavior is monostability, with one node at a high level of expression and the other node low^3^.

**Fig. 2:**
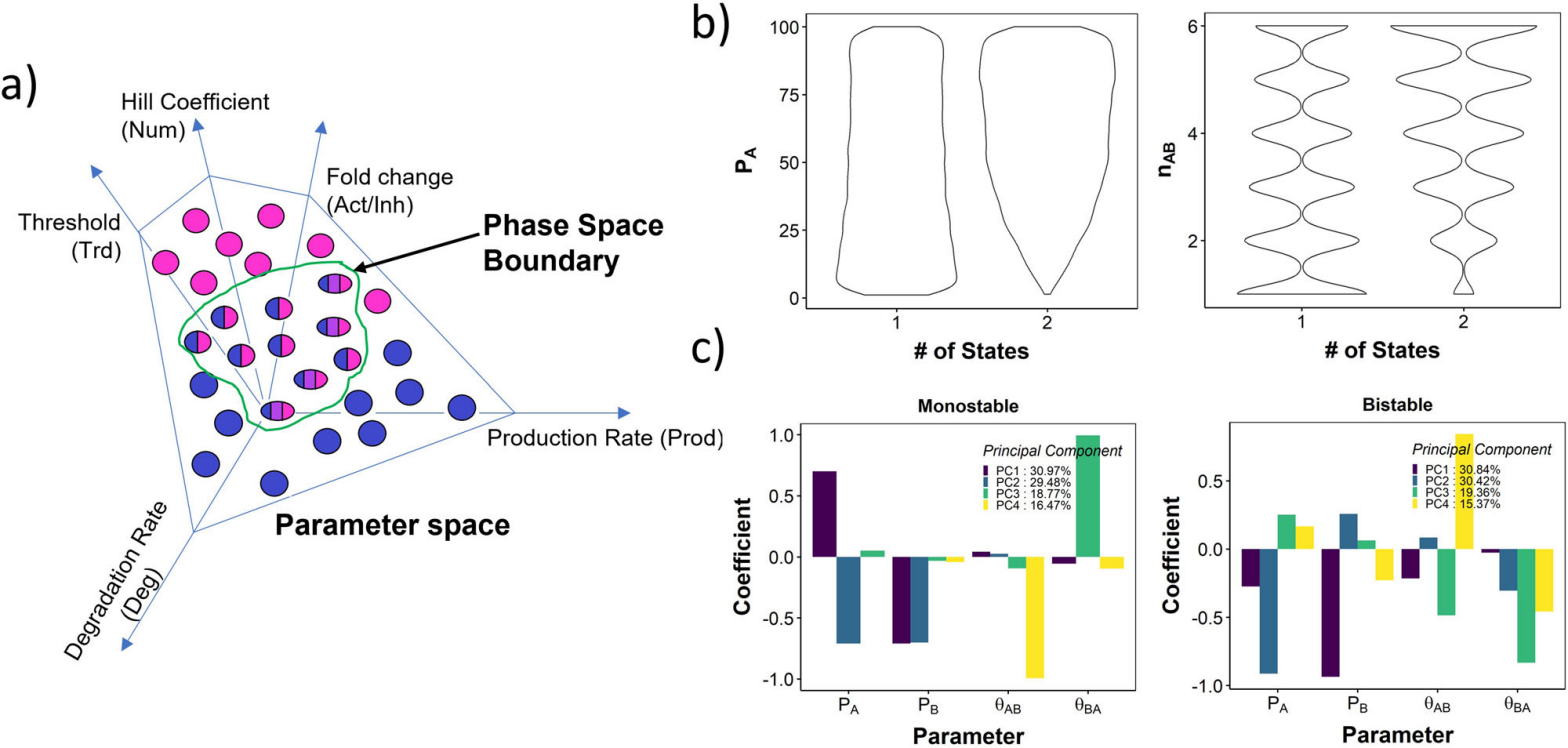
Identifying attractor-repertoire boundaries in RACIPE parameter space. **a** Depiction of attractor-repertoire space boundaries in RACIPE. The parameter space is defined by the ranges of the five types of parameters. Each sampled parameter set can be monostable (unicolored ovals) or multistable (multicolored ovals). The attractor-repertoire boundary (green closed curve) separates monostable parameter sets from multistable parameter sets. **b** Distribution of Production rate of A (left) and Hill coefficient of A → B link (right) for monostable and bistable parameter sets in toggle switch. **c** Barplot depicting the Principal component coefficients 1-4 for the four top parameters obtained from PCA of monostable (left) and bistable (right) parameter sets for toggle switch.

We then sought to understand the contribution of individual parameters in distinguishing between the categories of a given emergent behaviour. RACIPE samples five types of parameters (Fig. 2a). For TS, which has two nodes and two edges, the parameters are labelled as follows

- Node level parameters
  - *P*_*A*_ (*P*_*B*_): Production Rate of node A (or B)
  - *γ*_*A*_ (*γ*_*B*_): Degradation Rate of node A (or B)
- Edge level parameters
  - *i*_*BA*_: Inhibition fold change of the effect *A* on *B*
  - *a*_*BA*_: activation fold change of the effect *A* on *B*
  - *n*_*BA*_: Hill coefficient of the effect *A* on *B*
  - *θ*_*BA*_: Threshold for the effect of *A* on *B*.
  Analogous effects of *B* on *A* will have subscripts *AB*.

The details of the ODE system are given in Methods section. We first checked if the distributions of any of the individual parameters can delineate monostability from multistability. We find that while there are no clear delineators, the bistable parameters show a lower frequency of low Hill coefficient, low production rate, and high degradation rate (Fig. 2b, Supplementary Fig. 1).

Given the non-linearity of the system, it is understandable that no individual parameter can separate the monostable parameter sets from bistable parameter sets, implying that some combination of parameters together should be able to make the distinction between the two classes of parameter sets. To test this hypothesis, we then performed PCA separately on monostable and bistable parameter sets. In both cases, the first four axes of PCA together could explain > 95% variance (Fig. 2c, legend). At first glance, the primary contributors to the variance (highest coefficient in PCA axes) for both monostable and bistable parameter sets are the two production rates and two thresholds corresponding to the two edges in TS. Furthermore, in PC1 of the monostable parameter sets, the coefficients of the production rates have opposite signs, while the same coefficients in bistable parameter sets have the same sign. In PC2, the reverse pattern is observed, i.e., same sign in monostable parameter sets and opposite sign in bistable parameter sets. The threshold parameters had a stronger presence in PC3 and PC4 in both monostable and bistable parameter sets. However, while threshold parameters are exclusively limited to PC3 and PC4 in monostable parameter sets, they also contributed to PC1 and PC2 in bistable parameter sets. This indicates higher complexity of the description of the parameter set that exhibits bistability.

**Fig. 3:**
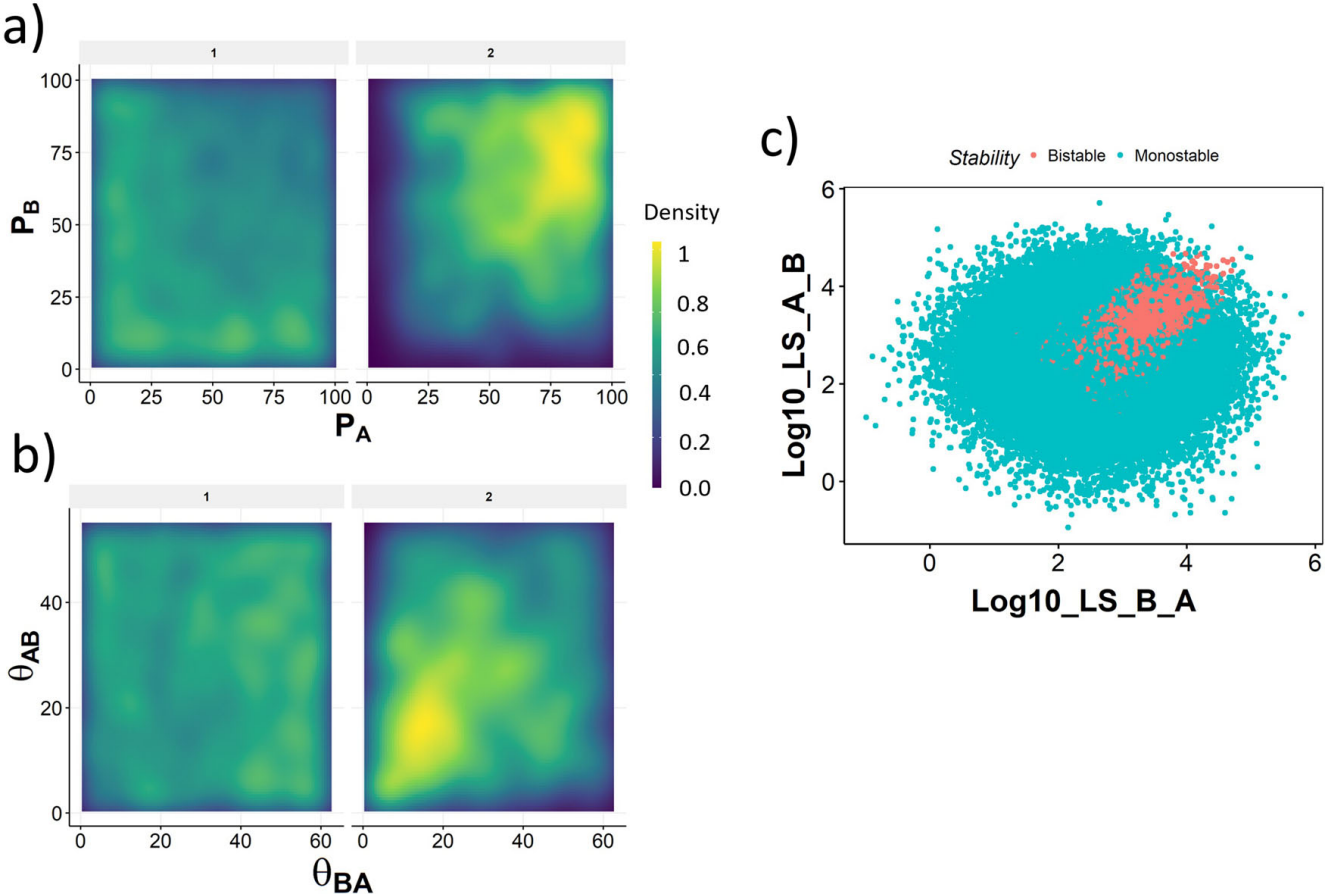
Link strength delineates monostable and bistable parameter sets. **a** Density distribution of the parameters Production rate of A and B for monostable (panel label - 1) and bistable (panel label - 2). The color scale varies from purple (low) to green (medium) to yellow (high). Higher density indicates higher number of parameters in the corresponding region. **b** Same as **a**, but for Thresholds for *A* → *B* and *B* → *A* links. **c** Scatterplot between the link strength values demonstrating the separation of parameter domains by link strength.

To better understand the patterns observed in PCA, we moved on to ask if the production rate and threshold value can delineate bistability from monostability. We generated a density plot of monostable and bistable parameters along the axes of both production rates, to identify the ranges of the production rates that are observed most frequently in mono and bistable cases. These plots indicated that while monostable parameter sets show higher incidence when two production rates are very different i.e. Prod_of_A ≫ Prod_of_B and Prod_of_B ≫ Prod_of_A, or when both production rates are low, in bistable parameter sets we see a higher incidence of approximately equal production rates Prod_of_A ≈ Prod_of_B (Fig. 3a). Furthermore, the production rates in bistable parameter sets had a higher chance of being above the median production rate (50) sampled by RACIPE. A similar visualization involving threshold parameters revealed that both threshold values tend to be lower than median and similar to each other in bistable parameter sets (Fig. 3b). No clear trend was observed for threshold in monostable parameter sets, suggesting that threshold values being smaller is necessary but not sufficient for bistability.

These patterns can be interpreted as follows: high production rate of A and low threshold from A to B implies a higher chance of the inhibition from A to B being active; similarly, a high production rate of B and low threshold from B to A implies a higher chance of the inhibition from B to A being active. Hence, we hypothesized that occurrence of bistability in TS requires both links to be simultaneously active. On the other hand, the emergence of monostability is associated with asymmetry of the production rates, where one of them is higher than the appropriate threshold, while the other one is lower than its threshold. Therefore we hypothesize that monostability occurs when one of the links is significantly stronger than the other, while bistability occurs when both links have similar and high strength.

To test this hypothesis, we adapted a measure of link strength^30^ to explain the emergence of certain phenotypes in GRNs. The link strength takes the following non-dimensional form:

1 $$L{S}_{AToB}=\frac{{P}_{A}* {n}_{BA}}{{\gamma }_{A}* {\theta }_{BA}* {i}_{BA}}$$

We generated a scatter plot of the log value of the link strengths on either axis (Fig. 3c). Interestingly, using a logarithm transformation on these two parameters converts the set of sampled parameters into a circular cloud where parameters that support bistability form a small section of the cloud. Note that this section is in the region that corresponds to both links being strong and similar in value, which is consistent with our hypothesis that both links should have similar and high strength for bistability.

We further tried to delineate different attractor-repertoires (10, 01, 01-10 etc) of mono- and bistability using link strength S2. Low link strength (<3) for both links leads to 00 attractor-repertoire. For a majority of the parameter sets displaying attractor-repertoire 10, link strength of A to B is low. Similarly for attractor-repertoire 01, link strength of B to A is low. However, we find it hard to distinguish between 11 and 01-10 attractor-repertoires using link strength analysis. Furthermore, for attractor-repertoires 10 and 01, there exist cases with high link strength for both links.

While link strength can delineate the monostable and bistable parameter sets better than any individual/combination of parameters tried so far, the boundary between bistability and monostability and that between individual attractor-repertoires is not completely sharp. This leads to uncertainty in prediction of multistability of a parameter for TS. Therefore we tried to clarify and sharpen this boundary through DSGRN.

### DSGRN inequalities define clear boundaries separating attractor-repertoires

DSGRN (Dynamic Signatures Generated by Regulatory Networks) is a modeling platform that assigns to a GRN a *switching ODE system* with undetermined parameters^4–7^. The parameters include a threshold value, as well as low and high production rates assigned to each edge and a decay rate assigned to each node. At each node, if the combination of high and low values transmitted along the incoming edges is higher than a particular threshold of an outgoing edge, this edge is activated. In the case where this is an activating edge, a high production rate is triggered; when there is a repressing edge, a low production rate is triggered. Using these parameters, DSGRN provides an explicit finite decomposition of the parameter space into parameter domains, such that for all parameters in each domain the state transition graph (STG) (see Methods section for detailed description), the attractor-repertoire is invariant.

The parameters of the switching ODEs that are used by DSGRN can be related to the parameters used by RACIPE formalism (Methods “Translating Shifted Hill functions to DSGRN”). We therefore asked if DSGRN inequalities can be used to delineate parameter spaces in RACIPE and predict the outcome of the dynamics at parameters sampled by RACIPE. As a first step, we carefully compared the structure of the ODE models used in RACIPE and DSGRN (see Methods).

There are two major differences between RACIPE and DSGRN. First, while RACIPE uses model equations with a finite Hill coefficient (sampled by default between 1 and 6), DSGRN uses piece-wise constant nonlinearities that can be obtained as a limit of Hill functions where the Hill coefficient *n* → *∞*. Second, in DSGRN the production rate for a node is calculated independently for each incoming edge, followed by combining these production rates together (taking a product) to get the net production rate of the node. In RACIPE, each node is assumed to have a basal production rate which gets multiplied by a fold change parameter for each incoming edge. Assuming that each incoming edge has an equal contribution to the basal production rate, we establish a one-to-one correspondence between the parameters of RACIPE and DSGRN, with the exception of the Hill coefficient. With this translation between these two approaches, we find that the link strength formalism described previously has a similar form as the inequalities obtained from DSGRN that define the parameter nodes.

DSGRN does not predict what attractor-repertoire will be attained for a given initial condition or specific real-valued parameter set. However, an explicit switching ODE system can be constructed that does and faithfully reflects the predictions of DSGRN, see Methods “Translating Shifted Hill functions to DSGRN”. This framework allows a direct comparison between a RACIPE simulation and the corresponding DSGRN prediction for a set of initial conditions and parameters for a given GRN. “Switching system” and “DSGRN” may be used interchangeably in what follows.

**Fig. 4:**
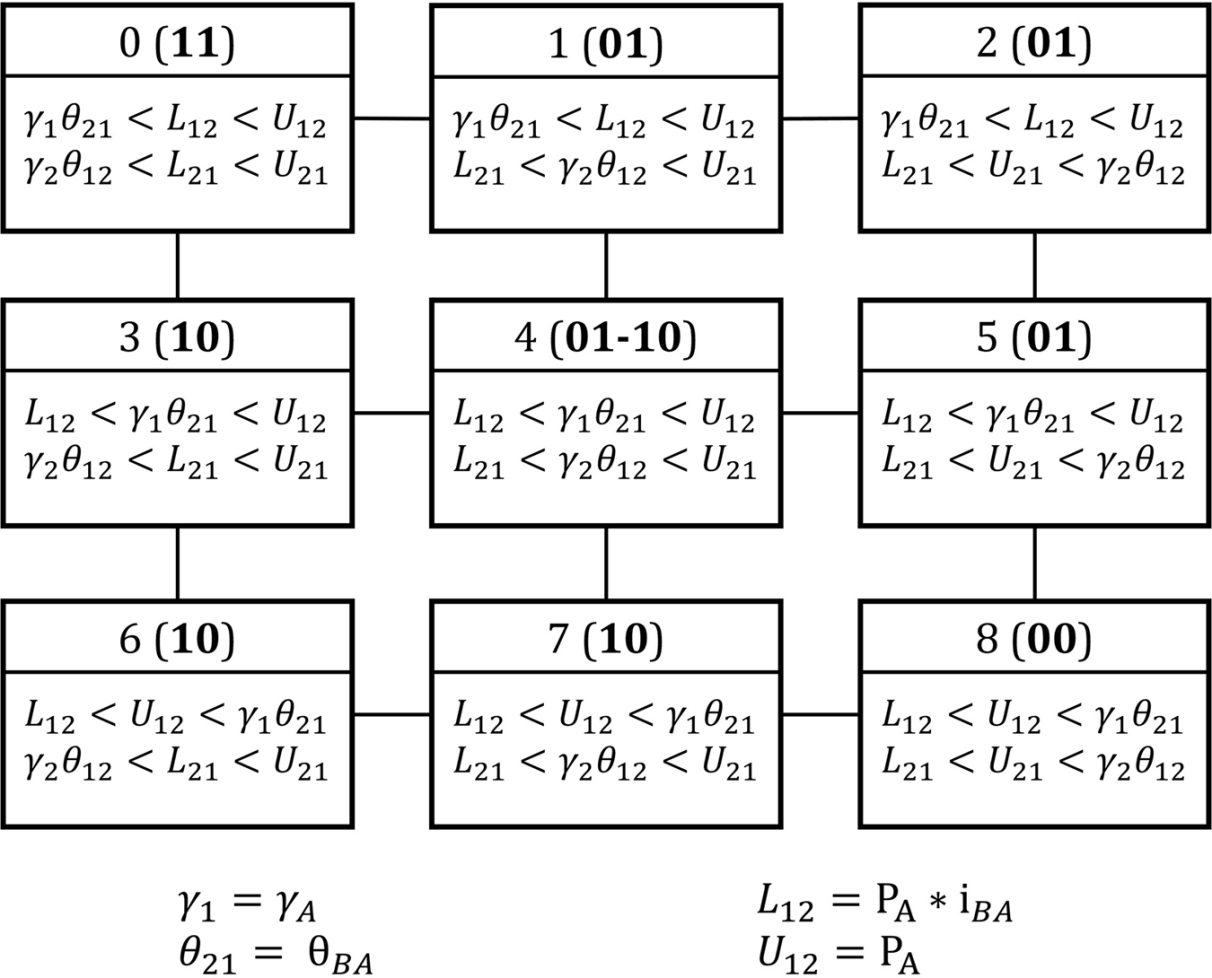
Depiction of the parameter domains/nodes calculated by the DSGRN inequalities for TS^41^. Each box represents a parameter node. The inequalities that define the parameter node are given in the lower part of the corresponding box. At the top of each box, the integer provides a reference to each parameter node, followed by the description of the attractor-repertoire at each parameter sample satisfying the inequalities, as predicted by DSGRN.

**Fig. 5:**
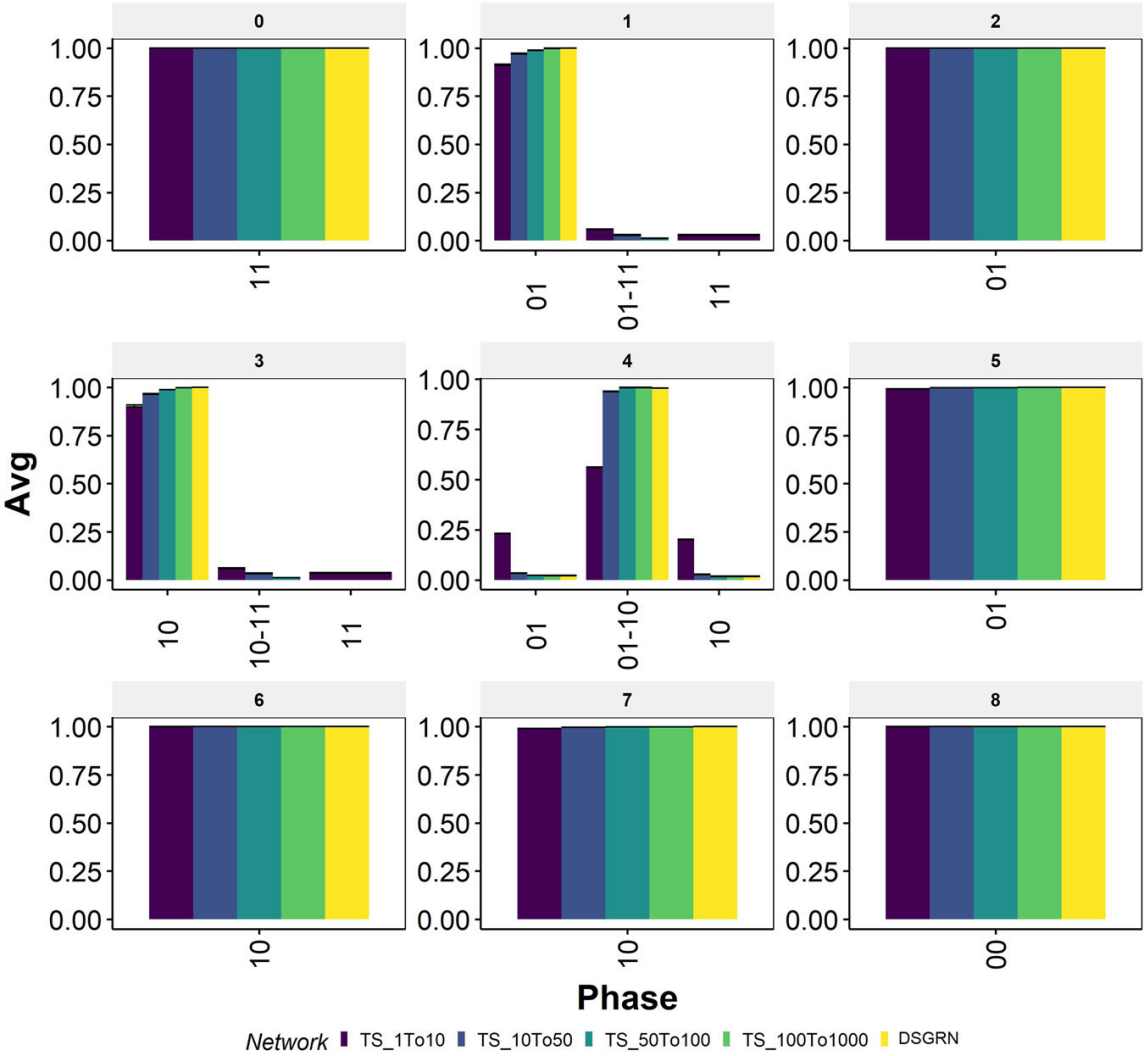
Attractor-repertoire distribution of the switching system against different ranges of Hill coefficients in RACIPE for TS. The switching system is represented by yellow colored bars. Default RACIPE conditions are represented by the dark blue colored bars. The range of Hill coefficients in each case is reported in the color-legend.

Using this correspondence we imposed the inequalities calculated in DSGRN onto RACIPE parameter sets, and obtained a attractor-repertoire distribution for each parameter domain, where a parameter domain is defined by a unique combination of inequalities (Fig. 4). Because DSGRN corresponds to a RACIPE model where the Hill coefficient *n* approaches infinity, i.e. for very steep Hill nonlinearities, we expect that as *n* gets larger, the correspondence between DSGRN prediction and RACIPE simluation will improve. However, this argument does not provide any information on the accuracy of this prediction for relatively small values of *n*. Somewhat surprisingly, most of the monostable parameter nodes show nearly identical behavior between RACIPE simulation and DSGRN prediction, even at relatively small values of Hill coefficient *n* ∈ {1,…,10}. However, at low values of *n* the central parameter node (parameter node 4, Fig. 4) shows larger discrepancies. For these sets of parameters DSGRN predicts bistability between steady states 01 − 10, i.e., any parameter set obeying the inequalities will exhibit bistability with the states being 10 and 01. RACIPE on the other hand shows a more heterogeneous attractor-repertoire distribution, with predominant attractor-repertoire still being bistable 01 − 10, but also registering monostable states 10 and 01 (Fig. 5 center panel, parameter node 4).

To check whether the increase in the Hill coefficient will bring the RACIPE simulations and DSGRN predictions closer together, we modified the RACIPE parameters by sampling Hill coefficient values uniformly from various ranges (1 − 10, 10 − 50, 50 − 100, 100 − 1000) and compared the parameter node-wise attractor-repertoire frequencies with those of DSGRN (Fig. 5). While the Hill coefficient range of 50 − 100 shows near identical results as that of DSGRN, even range of 10 − 50 is similar to DSGRN within reasonable error (<5%). Further probing of parameter node 4 in RACIPE revealed that, having both Hill coefficients greater than 5 is enough to get the frequency of bistable attractor-repertoire (10-01) to be greater than 90% (Supplementary Fig. 3).

We observed similar trends for the other two node network motifs: Double Activation (DA) and Negative Feedback loop (NF), (see Supplementary Fig. 4, 5, and 7). For both of these networks, RACIPE shows similar results as DSGRN at moderately high Hill coefficient values (>10). Therefore, DSGRN inequalities can be used to clearly delineate RACIPE parameter space into different attractor-repertoires. Furthermore, DA network shows bistability for parameter node 4 (FP(00-11)), which for some parameter sets is lost in RACIPE at lower values of Hill coefficient, but s recovered with increase in Hill coefficient values.

NF shows particularly interesting trends. The “frustration" in NF network (i.e., while A activates B, B inhibits A, causing the state of the system to oscillate^31^) leads to the prediction of cycles in DSGRN at parameter node 4. For the corresponding parameters, RACIPE at lower values of Hill coefficients (<10) predicts predominant monostability. We wanted to check if any of the parameters have been mislabelled as monostable while they are actually oscillatory, since RACIPE cannot identify oscillations. First, we confirmed whether the output of RACIPE is actually a steady state by imposing the condition that the derivative of all nodes should be small for steady states (see Methods section) We find that for all parameters predicted by DSGRN to be cyclic, the steady state obtained from RACIPE satisfied the derivative condition with a tolerance value of 10^−4^. Interestingly, as we increased the range of Hill coefficient to 10 − 50, RACIPE predicts all of these parameter sets to have ten steady states, which is the maximum number of steady states RACIPE can detect for a given parameter set. None of these states have a low derivative. Because DSGRN predicts cycles for these parameter sets, we categorised the parameter sets that show ten states in RACIPE, with none of them being a steady state, as cyclic.

The analysis so far suggests a transition from monostability to multistability (cyclic behavior for NF) in some RACIPE parameters as the Hill coefficient increases, a frequently observed pattern in GRNs^32^. This observation implies that the parameter sets that showed monostability at low Hill coefficients can acquire a new behavior as the Hill coefficient increases. These new behaviors will be more or less detectable numerically depending on the relative size of their basins of attraction. It is desirable to have a prediction not only of attractor-repertoire type and number, but also the relative sizes of their basins of attraction.

An analytical computation of the volume of the basins of the attraction in bounded region of attractor-repertoire space even for a simple system like TS is intractable due to nonlinearities. However, the boundaries of the basins of attraction for the TS switching system can be analytically computed (Methods section “Basin of attraction boundaries for bistable Toggle Switch”). Then any initial condition can be analytically associated to its long-term attractor-repertoire by its relation to the basin boundaries. The fraction of sampled initial conditions that go to a given attractor-repertoire is then an estimate of the relative basin size for that attractor-repertoire. There is no similar analytic boundary calculation for RACIPE, but the relative basin size can be numerically approximated by taking the fraction of initial conditions that converge to a given attractor-repertoire. We use these approximations to compare RACIPE basin sizes with various Hill coefficients to the limiting behavior in the switching system.

For comparison purposes, we defined a scalar “basin strength” to be the product of the fraction of initial conditions that converge to each detected attractor-repertoire. For monostability, this fraction can be at most 1 and for bistability it is maximally 0.25 (0.5*0.5 for equal basin sizes). We then calculated the difference between the RACIPE basin strength and DSGRN basin strength. The difference can range between −1 and 1, such that −1 is attained for parameter sets where RACIPE predicts monostability (basin strength 1) while DSGRN predicts an uneven bistability (i.e., low value of basin strength). Since the analysis is carried out for parameter sets belonging to parameter node 4, the basin strength for DSGRN will always be less than 0.25, making the upper limit for the difference 0.25.

In Fig, 6, we plot this difference for a collection of 10000 randomly chosen parameters for both high and low Hill coefficients. We observe that for a given parameter set, as the RACIPE Hill coefficients increase, the basin strength of RACIPE simulations get closer to that of DSGRN basin strength (left and middle panels). This means that DSGRN boundary computations can be used to predict the relative sizes of basins of attraction.

**Fig. 6:**
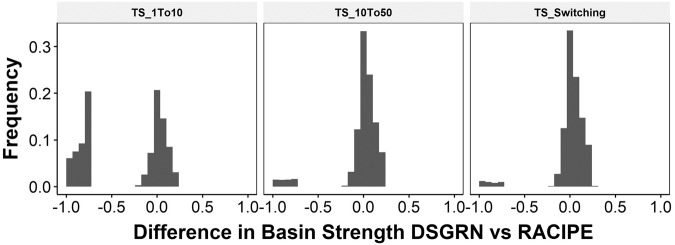
Switching system and RACIPE at high Hill coefficient show similar basin strengths as DSGRN.

In larger networks for which analytical boundary computations in DSGRN are infeasible, an estimation of basin of attraction size for the switching system can be made analogously to RACIPE by numerically computing the fraction of initial conditions that converge to each attractor-repertoire. In Fig. 6 (right), we compare the analytical assignment of attractor-repertoire via basin boundary to this numerical approximation. We see that such a switching system approximation identifies the correct attractor-repertoire in about 95% of cases, making the contribution of the numerical simulations to the error in prediction about 5%.

### Similarity between RACIPE and DSGRN holds for Toggle Triad

**Fig. 7:**
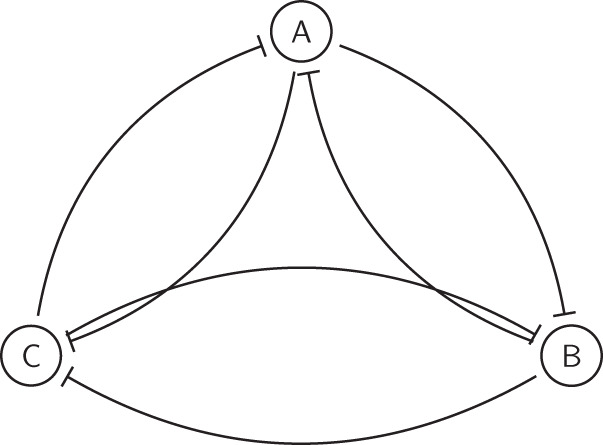
Toggle Triad (TT) network structure.

So far, we have found that in all three two-node networks that we studied, there is close correspondence between RACIPE and DSGRN. This suggests that DSGRN inequalities are able to describe how the dynamical behavior of the RACIPE model depends on parameters and delineate parameter regions of different attractor-repertoires. We now examine if these results hold for larger networks. To do this we chose Toggle Triad (TT), a three node network that can be viewed as a coupling of three toggle switches with two embedded negative feedback loops (see Fig. 7). The most interesting feature of TT is that it can exhibit tristability (High-Low-Low, Low-High-Low, Low-Low-High).

**Fig. 8:**
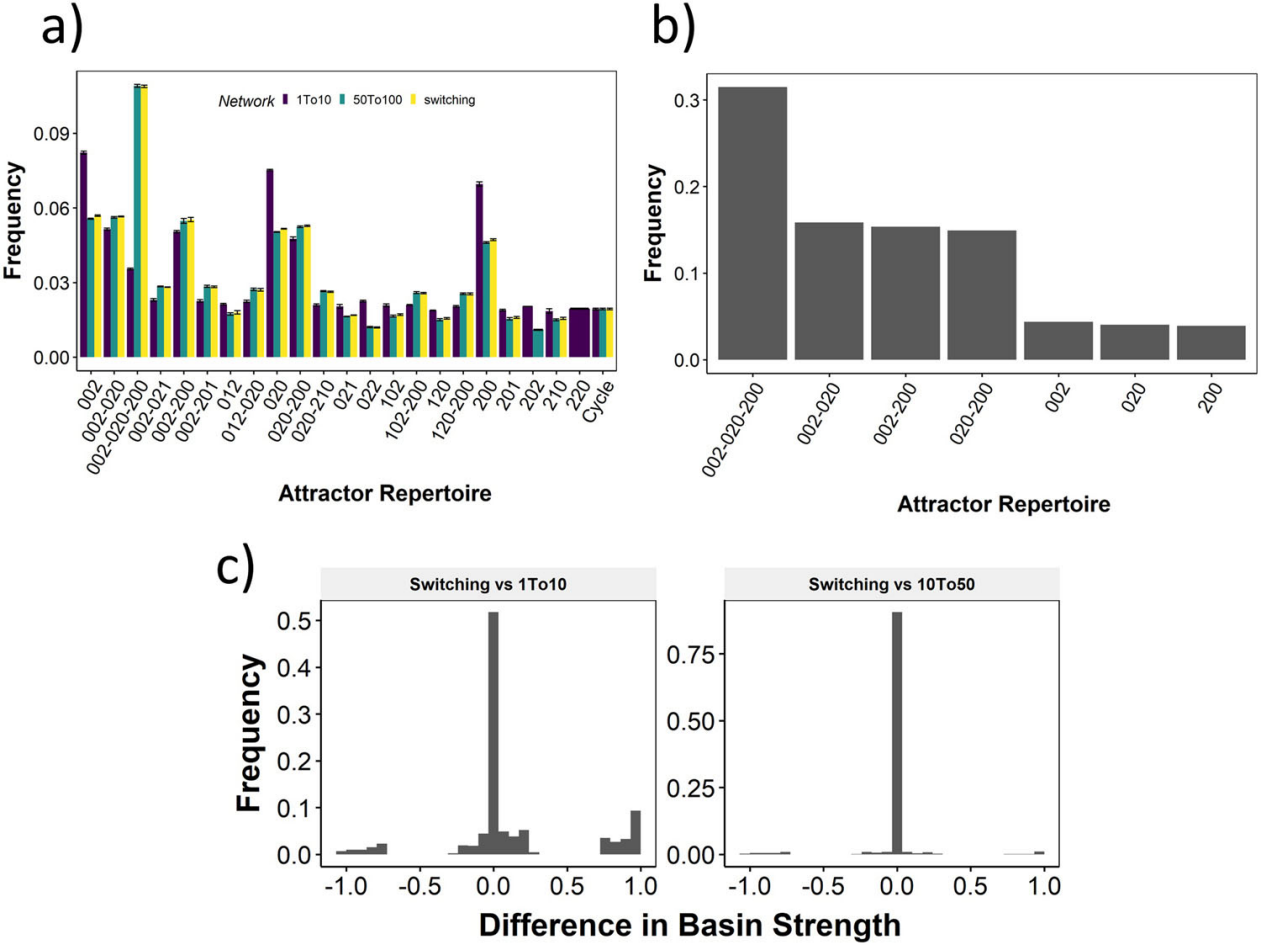
Comparison of RACIPE and DSGRN for Toggle Triad. **a** Bar graph depicting the change in mean frequency of attractor-repertoire occurrence for RACIPE with Hill coefficient between 1 and 10 (purple), 50 and 100 (green) and switching system (yellow). The error bars represent mean ± standard deviation. **b** attractor-repertoire frequency distribution for parameter sets that exhibit tristability in switching system in RACIPE model with *n* ∈ [0, 10]. **c** The difference in basin strengths for switching system and RACIPE with low Hill coefficients (right) and RACIPE with high Hill coefficients (left).

Since each node in a toggle triad has two inputs, the number of DSGRN parameter nodes is much higher than that for the two node networks. Therefore, instead of focusing on a particular parameter node, we decided to study the distribution of attractor-repertoires across RACIPE parameters sample sets. In Fig. 8a, we compare attractor-repertoire predictions at each parameter given by RACIPE simulations to the predictions given by switching system simulations (equivalent to predictions by DSGRN). As with the two node networks, the similarity between RACIPE and DSGRN predictions increases with increasing Hill coefficient. We further probed the similarity between the predictions in terms of the frequency of attractor repertoire for a moving window of Hill coefficient ranges (Supplementary Fig. 6). As a control case, we first looked at the TS network, where we previously observed that Hill coefficients of 5 could lead to a similar attractor-repertoire distribution as that of DSGRN (i.e., most parameter sets corresponding to parameter node 4 converge to 10-01 attractor-repertoire) (Fig. 5). The Jensen-Shannon Divergence (JSD)^33^ between the attractor repertoire frequency distributions saturates after a Hill coefficient of 7, indicating maximum similarity befigtween DSGRN and RACIPE (Supplementary Fig. 6a). For TT, this saturation is observed beyond a Hill coefficient of 20 (Supplementary Fig. 6b). Furthermore, the absolute JSD values are significantly higher than that observed for the toggle switch. We repeated similar analysis for larger networks having four nodes. The toggle square is a cyclic chain of four toggle switches connected end-to-end (i.e, A to B, B to C, C to D and D to A), thereby having eight edges. The GRHL2 network is a biological network^22^ with seven edges. Both these networks showed a higher JSD value, which is clearly visible at the limit of high Hill coefficients (Supplementary Fig. 6c, d). The formalisms of DSGRN and RACIPE are directly related as shown before (Methods Section “Translating Shifted Hill functions to DSGRN”), and thus JSD must converge to zero in the limit of large Hill coefficient. Hence, we attribute this increase in JSD to the accumulation of numerical errors in the simulation, caused by the use of Euler method of integration and other factors. A numerically accurate implementation of RACIPE should hence improve the similarity between the distributions of attractor-repertoire distribution as the relation between the two formalisms suggests.

Taking a closer look at the attractor repertoires predicted by RACIPE and DSGRN for Toggle Triad, we see a higher frequency of tristable attractor-repertoire 002-020-200 in DSGRN and in RACIPE with high Hill coefficients compared to RACIPE with lower Hill coefficients. Among the parameter sets that are tristable in DSGRN, greater than 30% of them show tristability at low Hill coefficients in RACIPE. Of the remaining 70%, more than half show bistability equally distributed between 002-020, 002-200 and 020-200, which are all different subsets of the tristable attractor-repertoire, see Fig. 8b. To eliminate the possibility that at low Hill coefficients, the tristability is not detected due to a smaller number of initial conditions, we simulated these networks for an increased number of initial conditions. We found that for 1000 and 10000 initial conditions, we do not observe the missing steady states for any initial condition. The difference in basin strengths also vanishes between the switching system and RACIPE (Fig. 8c), suggesting that these parameter sets that showed bistability at low Hill coefficient gain another steady state as *n* increases.

### Contribution of the parameter sampling in RACIPE to the differences in RACIPE and DSGRN

**Fig. 9:**
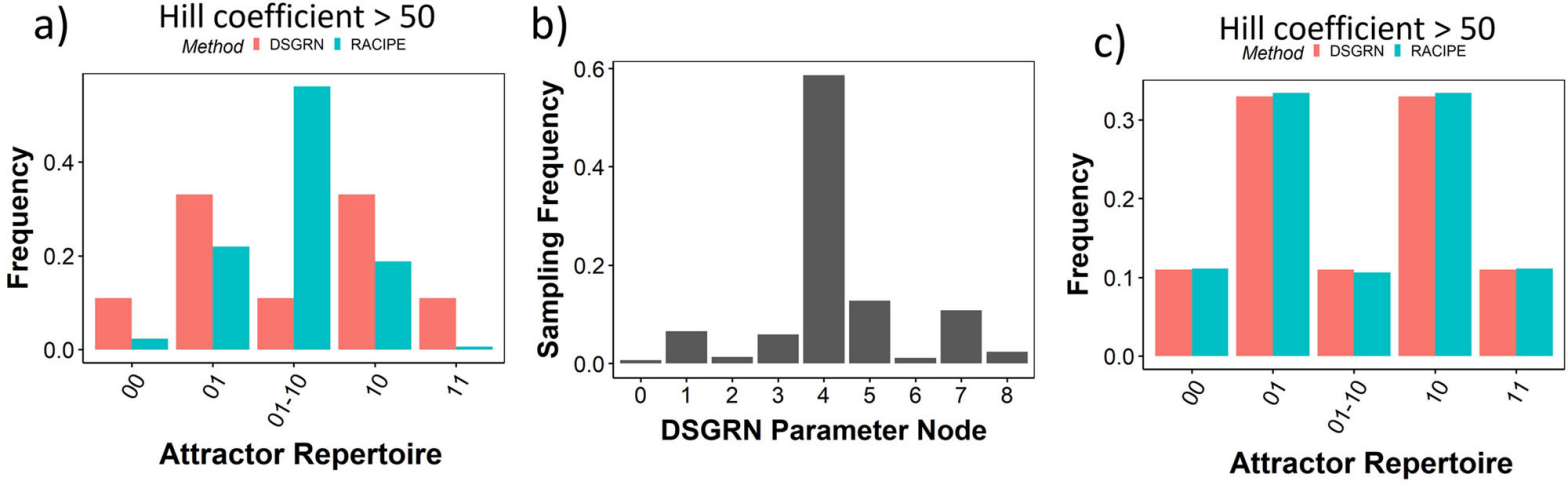
RACIPE shows uneven sampling of DSGRN parameter nodes. **a** Comparision of attractor-repertoire frequency distributions for RACIPE with high Hill coefficient (green) and DSGRN (red). **b** Distribution of DSGRN parameter nodes sampled by RACIPE. **c** Same as **a**, but RACIPE attractor-repertoire frequencies are normalized by number of samples from the corresponding DSGRN parameter domains.

Both RACIPE and DSGRN are capable of predicting the ensemble level behavior of a GRN. Given the similarities between RACIPE and DSGRN formalisms at high Hill coefficient, we compared the ensemble distributions, i.e. frequency distribution of individual attractor-repertoires obtained across all sampled parameter sets for RACIPE and all parameter nodes for DSGRN for the 2-node networks TS, DA and NF. Unlike the comparisons across individual parameter nodes, the ensemble frequency distribution of RACIPE predicted a higher frequency for the 01 − 10 attractor-repertoire as compared to DSGRN (Fig. 9a). As the differences in the model formalism should diminish at high Hill coefficient values, we looked at the distribution of parameters sampled by RACIPE with respect to the DSGRN parameter domains. Interestingly, we find that RACIPE’s sampling method is highly biased towards the parameter node 4, which explains the prediction of high bistability from RACIPE at high Hill coefficient Fig. 9b). Importantly, while DSGRN parameter domains decompose the parameter space into disjoint number of parameter domains (for TS, DA and NF there are 9 domains), this method is agnostic on where the biologically relevant parameters lie. If we assume that the RACIPE methodology samples biologically relevant parameters, then the non-uniform distribution of samples may be taken as a hypothesis which of the parameter domains are more important. The results here suggest, that even though only 1 out of 9 DSGRN parameter domains supports bistability, this domain is sampled by more than 50% of RACIPE parameters and hence it is more important than the ratio 1/9 would suggest. After normalizing the attractor-repertoire frequencies by the number of RACIPE parameters in each parameter node, the ensemble frequency distribution does match DSGRN predictions (Fig. 9c).

With the default range of Hill coefficients 1 − 6, RACIPE’s ensemble distribution is much closer to that of DSGRN (Supplementary Fig. 7). We have seen before that RACIPE samples the bistable/cyclic parameter sets (parameter node 4) with higher frequency relative to the other parameter nodes of DSGRN. However, at low Hill coefficients, this bistability/cyclicity deteriorates to monostability for a significant fraction of parameter sets belonging to parameter node 4. This deterioration serves to compensate for the biased sampling RACIPE conducts, thereby making the ensemble attractor-repertoire distribution of RACIPE at Low Hill coefficient similar to the corresponding DSGRN distribution.

## Discussion

Modeling gene regulatory networks is qualitatively different than modeling physical systems like those in celestial mechanics and ecology^34^. While the behavior of the latter is described by equations stemming from physical laws and whose parameters are well-defined measurable physical quantities, the models of GRNs describe dynamics on a spatial scale where the first principle models do not apply. For this reason, neither the functional form is fixed, nor the parameters are known. The parameter measurement for such descriptive models is challenging since the parameter is often only defined in the context of the model. Given these challenges, it is imperative to devise modeling approaches that deal with parameter uncertainty and describe the dynamics of the GRN in a way that describes the diversity of GRN dynamics across parameters.

In this paper, we compare two such complementary approaches. On one hand, RACIPE^3^ judiciously samples parameters for a reasonable Hill function model of GRN and uses this ensemble as a description of network dynamics. On the other hand, the DSGRN^4^ description of the parameter space using all collections of monotone Boolean functions compatible with the network dynamics can be related to switching ODE models and allows precise descriptions of parameter regions with given dynamics by explicit inequalities. Each of these approaches has its limitations. Sampling parameters in high dimensional parameter spaces may miss some dynamics and RACIPE does not come with a guarantee of completeness of represented dynamics in the set of samples. On the other hand, DSGRN can be directly linked only to models with very high Hill coefficient *n* → *∞*, which are not biologically realistic. However, DSGRN inequalities do give a detailed functional/phenotypic description of the parameter space, which is lacking in RACIPE. For a given GRN, DSGRN is able to clearly separate the boundaries of the parameter domains, where each domain defines a unique phenotype (mono/bistability, nature of the steady states obtained) emergent from the GRN. Hence, measuring the applicability of such descriptions to RACIPE could lead to an efficient mapping of the phenotypes to the corresponding parameter regimes of biological systems governed by GRNs.

We compared these methods to see if we can combine their advantages and mitigate their disadvantages. We established a translation between RACIPE and DSGRN models, allowing us to categorize RACIPE sampled parameter sets into DSGRN parameter domains. As expected, we find very good agreement between the phenotypes obtained from DSGRN and RACIPE with high Hill coefficients. Somewhat surprisingly, we also find a very good agreement for low ranges of *n* ∈ [1, 6], specifically in predicting monostability. The bistable parameter nodes from DSGRN always exhibited a mix of mono- and bistability in RACIPE. We further found that RACIPE’s sampling is strongly biased towards multistable/oscillatory parameter domains of DSGRN. Despite the differences, we found a striking similarity between the default RACIPE phenotypic distribution and that obtained from DSGRN. This agreement suggests that one can use explicit combinations of parameters supplied by DSGRN to predict emergent phenotypes for ODE models with realistic Hill coefficients. While numerical differences exist between these two methods at the limit of high Hill coefficients, we predict that these differences are a product of numerically inaccurate implementation of the RACIPE formalism. Inspired by this, we are working towards a better implementation of RACIPE without the loss of computational efficiency provided by the current formalism, to be reported and analyzed in future works.

In this paper we compare RACIPE and DSGRN for relatively small networks, both because parameter sampling of large networks likely does not cover all dynamic behavior, and the number of DSGRN parameter domains grows rapidly. However, recent theoretical work^18^ shows that for a network of any size, the stable equilibria in DSGRN examined in this paper perturb to stable equilibria of models with sufficiently steep continuous nonlinearities, which include Hill models^18^. Furthermore, for particular perturbations of DSGRN to ramp nonlinearities (i.e. constant functions joined by linear interpolation) one can explicitly estimate how shallow the nonlinearity can be while retaining DSGRN equilibria. Similar results were noted using the HillCubes approximation^27^ of Boolean functions encoded in the software Odefy^28^.

An important message from this study is the contribution each of the methods can make to enhance the capability of the other. Switching systems, being an approximation of DSGRN, can identify the possibility of multistability for parameter sets with higher accuracy than RACIPE, along with the nature of the states/phenotypes. The approximation of DSGRN output by switching systems is especially useful for networks of higher complexity, where the computation of DSGRN parameter nodes can be computationally expensive.

## Methods

### Random Circuit Perturbation (RACIPE)

RACIPE^3^ is a tool used to simulate continuous dynamics of gene regulatory networks (GRNs). For a given GRN, RACIPE constructs a set of ODEs representing the interactions in the network. For a node *k*, let *A*_*k*_ and *I*_*k*_ denote the set of all activating and inhibiting input nodes to *k* respectively and *x*_*k*_ denote the expression of node *k*. The dynamics of node *k* is given by the ODE

2 $$\frac{d{x}_{k}}{dt}={P}_{k}\mathop{\prod}\limits_{j\in {A}_{k}}\frac{{H}^{s}\left({x}_{j},{\theta }_{kj},{n}_{kj},{a}_{kj}\right)}{{a}_{kj}}\mathop{\prod}\limits_{j\in {I}_{k}}\,{H}^{s}\left({x}_{j},{\theta }_{kj},{n}_{kj},{i}_{kj}\right)-{\gamma }_{k}{x}_{k}$$

where for node *k*, *θ*_*kj*_ is the threshold value for the edge from *j* to *k*, *P*_*k*_ is the production rate, *γ*_*k*_ is the degradation rate, *n*_*kj*_ is the Hill coefficient, *i*_*kj*_ < 1 and *a*_*kj*_ > 1 are the fold changes for the inhibitory and activation edges, respectively. The function *H*^*s*^ is called the shifted Hill function, given by

3 $${H}^{s}\left(x,T,n,\lambda \right)=\lambda +\left(1-\lambda \right)\frac{{T}^{n}}{{T}^{n}+{x}^{n}}.$$

RACIPE simulates this set of ODEs by uniformly sampling parameter sets from a pre-determined range of parameters. These parameter ranges were estimated from BioNumbers^35^. For each parameter set, the ODEs are simulated using the Euler method for multiple initial conditions. The parameters used for each set of simulations and the stable states obtained towards which individual trajectories converge are recorded as an output of the simulation. We then verify the stability of the outputs via the following condition:

4 $$\sqrt{\frac{{\sum }_{k\in \{1,...,N\}}{\frac{d{x}_{k}}{dt}}^{2}{| }_{{x}_{k}^{* }}}{N}} \,<\, 1{0}^{-4}$$

where *N* is the number of nodes in the network.

### Processing RACIPE data

For each parameter set sampled by RACIPE, the different initial conditions generated converge to potentially several steady states. Each steady state obtained from RACIPE is assigned a weight, equal to the fraction of initial conditions that converge to the steady state for the corresponding parameter set. This results in a *M* × (*N* + 2) table, where *N* is the number of nodes in the network and *M* is the number of steady state-parameter set combinations. Since each parameter can have more than one steady state, *M* > = *N*. The first column showing the parameter ID, next *N* columns showing the expression level of each node of the network and the last column showing the weight for the corresponding steady state. The node expression values are converted to weighted z-scores by scaling them about their means:

5 $${z}_{j}=\frac{{x}_{j}-\bar{{x}_{j}}}{\sigma }$$

where the steady state expression vector of a node *j* across all steady state-parameter combinations is given by by *x*_*j*_, $\bar{{x}_{j}}$ is the weighted mean of the steady state expression of node *j*, and *σ* is the weighted standard deviation of the expression level. The weighted z-scores are then binarised by assigning a value of 1 for positive and 0 for negative weighted z-scores respectively. Hence, each steady state is encoded as a string of zeroes and ones of length that is the the number of nodes in the network.

### DSGRN description

A regulatory network summarizes the activating and repressing effects of molecular species on each other. A classical way to model the dynamics associated to a regulatory network is to introduce an ODE system for the concentrations of the various molecular species. A common model is called a *switching system*, in which each regulation event is regarded as a discontinuous and instantaneous switch when a concentration crosses a threshold. These systems are an approximation of the ODE model with graded responses but admit a rather more comprehensive analysis of its solutions^11,13,14,36–38^.

All ODE systems are dependent on a collection of parameters. In^4^, we showed that switching systems induce a finite decomposition of parameter space into semi-algebraic regions. Each region represents a *DSGRN parameter* that contains all the information needed to construct a *state transition graph* (STG) that captures a coarse description of the dynamics in attractor-repertoire space of the switching system. This graph is finite, and it is the same for all parameters in the region represented by the DSGRN parameter. This means that the analysis of the dynamics of the switching system over all of the parameter space is computable.

The DSGRN approach collects all of the combinatorial parameters into a graph and computes the STG for each on^4^ A condensed version of the STG called a *Morse graph* is a summary of the global structure of the dynamics for each combinatorial parameter. A *Morse node* of the Morse graph is a node representing a strongly connected path component or recurrent component of the STG, and it can be annotated with summary information about the component. We now describe these concepts in greater detail.

A *regulatory network* RN = (*V*, *E*) is a graph with network nodes *V* = {1, 2, …, *n*} and signed, directed edges *E* ⊂ *V* × *V* × { → , ⊣}. For *i*, *j* ∈ *V*, we will use the notation (*i*, *j*) ∈ *E* or *i* ⊸ *j* to denote a directed edge from *i* to *j* of either sign; *i* → *j* will denote an *activation* or positive interaction, and *i* ⊣ *j* will denote a *repression* or negative interaction.

We define the *targets* of a node *i* as **T**(*i*) ≔ {*j*∣(*i*, *j*) ∈ *E*} and the *sources* of a node *i* as **S**(*i*) ≔ {*j*∣(*j*, *i*) ∈ *E*}. 

A switching system takes the form

6 $${\dot{x}}_{i}=-{\gamma }_{i}{x}_{i}+{{{\Lambda }}}_{i}({\sigma }_{i1}^{\pm }({x}_{1}),\ldots ,{\sigma }_{in}^{\pm }({x}_{n}))$$

where each argument of Λ_*i*_ is either an increasing or decreasing step function, *σ*^+^ and *σ*^−^ respectively, defined by

7 $${\sigma }_{ij}^{+}({x}_{j})=\left\{\begin{array}{cc}{U}_{ij}&\,{{\mbox{if}}}\,{x}_{j} > {\theta }_{ij},\\ {L}_{ij}&\,{{\mbox{if}}}\,{x}_{j} < {\theta }_{ij},\end{array}\right.\quad {\sigma }_{ij}^{-}({x}_{j})=\left\{\begin{array}{cc}{U}_{ij}&\,{{\mbox{if}}}\,{x}_{j} < {\theta }_{ij},\\ {L}_{ij}&\,{{\mbox{if}}}\,{x}_{j} > {\theta }_{ij},\end{array}\right.$$

where 0 < *L*_*ij*_ < *U*_*ij*_ represent *lower* and *upper* activation (repression) levels of gene *i* by gene *j* and *θ*_*ij*_ is the *threshold* for the regulatory activity of gene *i* induced by gene *j*. Note that ${\sigma }_{ij}^{+}$ mediates an up-regulation of *i* by *j*, while ${\sigma }_{ij}^{-}$ mediates a down-regulation. We assume that all thresholds *θ*_*ij*_ are distinct, which is a generic assumption. The functions Λ_*i*_(*y*_1_, …, *y*_*n*_), *i* = 1, …, *n* are of the form

8 $${{{\Lambda }}}_{i}({y}_{1},\ldots ,{y}_{n}):= \mathop{\prod}\limits_{j}({y}_{{i}_{1}}^{\,j}+{y}_{{i}_{2}}^{\,j}+\ldots +{y}_{{i}_{k}(j)}^{\,j}),$$

where each *y*_*s*_ occurs exactly once. Such a function is *multi-linear* i.e. linear with respect to each *y*_*i*_, and has all coefficients equal to 1.

The collection of non-negative numbers *γ*_*i*_, *θ*_*ij*_, *L*_*ij*_, *U*_*ij*_ parameterizes the collection of systems (6). Let ${{{\mathcal{P}}}}$ be the collection of *regular* parameters, i.e. those that satisfy

9 $${\gamma }_{i}{\theta }_{ij}\,\ne \,{{{\Lambda }}}_{i}(\cdot )$$

for all (*i*, *j*) ∈ *E*. Since Λ_*i*_( ⋅ ) has only finite number of values, ${{{\mathcal{P}}}}$ is a generic subset of all non-negative parameters.

### State transition graph

The ordinary differential equation (ODE) system (6) admits a discrete description of attractor-repertoire space that is a directed graph called the *state transition graph*. The assumption that thresholds are distinct implies that each variable *x*_*i*_ has ∣*T*(*i*)∣ thresholds. The continuous attractor-repertoire space *R*^*n*^ consists of ${{{\Pi }}}_{i = 1}^{n}(| {{{\bf{T}}}}(i)| +1)$ domains *D*(*α*) where *α* is a multi-index with *α*_*i*_ ∈ {0, 1, 2, …, ∣**T**(*i*)∣}. Let

10 $$X=\{\alpha \ | \ \alpha =({\alpha }_{1},\ldots ,{\alpha }_{n}),{\alpha }_{i}\in \{0,1,2,\ldots ,| {{{\bf{T}}}}(i)| \}\}$$

be a generalized hypercube consisting of nodes with labels *α*. Each node represents a corresponding domain *D*(*α*) ⊂ *R*^*n*^.

We construct a state transition graph (STG) on *X* using the fact that solutions in each domain *D*(*α*) converge toward its associated *target point*$T\left.(\alpha )\right)=\left({{{\Lambda }}}_{1}(\alpha )/{\gamma }_{1},\ldots ,({{{\Lambda }}}_{n}(\alpha )/{\gamma }_{n})\right.$. The STG is a graph representation of the *asynchronous update* of the discrete-valued function Λ: *D*(*α*) → *T*(*α*). This means that the STG is a graph on *X* with edges between nodes *α*, *β* with ∣*α* − *β*∣≤1 assigned in the following way

- if *T*(*α*) ∈ *D*(*α*) then there is a self-edge *α* → *α*;
- if ∣*α* − *β*∣ = 1, *α*_*i*_ = *β*_*i*_ − 1 and the threshold between *D*(*α*), *D*(*β*) is *θ*, then *α* → *β* if *T*_*i*_(*α*) > *θ*; and *β* → *α* if *T*_*i*_(*β*) < *θ*.

Note that we can view the STG as a multivalued map ${{{\mathcal{F}}}}:X\rightrightarrows X$. The DSGRN approach^4–6,20,39,40^ compresses the information about the dynamics of ${{{\mathcal{F}}}}$ by computing the strongly connected path components of the STG and the reachability conditions between them. The strongly connected path components are the nodes of a *Morse graph*, which is a Hasse diagram of a partial order imposed by the reachability in the STG. For the purposes of this paper we will be most concerned with Morse nodes that represent a single node in the STG (and thus a single domain in the attractor-repertoire space) where all edges point inwards. This signifies existence of a stable steady state for the ODE (6) which will be denoted as *FP*(*β*) where *β* ∈ *H* determines osition of this steady state with respect to thresholds in the attractor-repertoire space. For instance for the toggle switch, which has one thresholds in each component the space *H* = {0, 1} × {0, 1} and thus the fixed points *FP* can have signatures (00), (01), (10), (11). 

### Parameter space decomposition

The most important construction is the decomposition of the regular parameter space ${{{\mathcal{P}}}}$ into a finite set of domains, such that for all parameters *p* in one of these domains, the STG is the same.

Let **RN** = (*V*, *E*) be a regulatory network and let $p\in {{{\mathcal{P}}}}$. Then for every *i*, every *j*_*n*_ ∈ **T**(*i*) and every domain *D*(*α*) exactly one of the following inequalities holds

11 $${{{\Lambda }}}_{i}(\alpha ) \,<\, {\gamma }_{i}{\theta }_{{j}_{n},i}\quad \,{{\mbox{or}}}\,\quad {{{\Lambda }}}_{i}(\alpha ) \,>\, {\gamma }_{i}{\theta }_{{j}_{n},i}.$$

This collection of abstract inequalities, together with the threshold order ${\theta }_{{j}_{1},i} < ... < {\theta }_{{j}_{| T(i)| },i}$ defined by *p* defines a region ${{{\mathcal{R}}}}$ of parameter space where every $p\in {{{\mathcal{R}}}}$ induces the same set of inequalities. We call such a collection of inequalities a *DSGRN parameter*.

We can organize domains of parameters in the form of a parameter graph. with a node in a parameter graph (PG), we complete the construction of PG by assigning edges to pairs of nodes that correspond to domains that share a codimension-1 boundary in ${{{\mathcal{P}}}}$.

Given **RN** = (*V*, *E*) we represent each DSGRN parameter as a *parameter node* in a DSGRN *parameter graph* **PG**. Two nodes are connected by undirected edge if there is a single inequality change between the corresponding collection of inequalities.

The parameter graph is a product of undirected *factor graphs*, **PG** = Π_*i*_**PG**_*i*_, one associated to each vertex *i* ∈ *V*. The class of possible factor graphs is determined by the topology of network node *i* and the algebraic expression Λ_*i*_^4^.

Given a node ${{{\mathcal{N}}}}\in {{{\bf{PG}}}}$, each $p\in {{{\mathcal{N}}}}$ defines the location of the target points for each of the domains *D*(*α*). Importantly, this location is independent on choice of $p\in {{{\mathcal{N}}}}$. Therefore the state transition graph and Morse graph can be associated to a parameter node ${{{\mathcal{N}}}}\in {{{\bf{PG}}}}.$

While for a precise general definition of the parameter graph we refer the reader to^4,5,39^, we will describe here its construction for the 2-node network examples needed in this paper. For node *i* with one input *i* − 1 and one output *i* + 1, the value of Λ_*i*_ is either *L*_*i*,*i*−1_ or *U*_*i*,*i*−1_. Then there are three choices

12 $${\gamma }_{i}{\theta }_{i+1,i}\, <\, {L}_{i,i-1} \,<\, {U}_{i,i-1},\qquad {L}_{i,i-1} \,<\, {\gamma }_{i}{\theta }_{i+1,i} \,<\, {U}_{i,i-1},\qquad {L}_{i,i-1}\, <\, {U}_{i,i-1} \,<\, {\gamma }_{i}{\theta }_{i+1,i}$$

that form the parameter factor graph *PG*_*i*_. Note that in all the cases of Toggle Switch, Dual Activation, and Negative Feedback loop there are two nodes in the network and both nodes have one input and one output. Therefore the parameter graph is a product *PG* = *PG*_1_ × *PG*_2_ with 9 nodes depicted in Fig. 4. Note that in the horizontal direction, i.e along the *PG*_1_ in the product, the inequalities (12) with *i* = 1 are varying, while in the vertical direction along *PG*_2_ the inequalities (12) with *i* = 2 are changing.

### Basin of attraction boundaries for bistable Toggle Switch

In this section we derive analytical expression for stable manifolds of the saddle point in TS based on a switching ODE system.

We consider switching system model of TS with equal decay rates. If we need more general formulation, the below argument can be modified. The model is

13 $$\begin{array}{ll}\dot{x}&=-{\gamma }_{x}x+\left\{\begin{array}{ll}{L}_{xy}&y \,>\, {\theta }_{xy}\\ {U}_{xy}&y \,<\, {\theta }_{xy}\end{array}\right.\\ \dot{y}&=-{\gamma }_{y}y+\left\{\begin{array}{ll}{L}_{yx}&x \,>\, {\theta }_{yx}\\ {U}_{yx}&x\, <\, {\theta }_{yx}\end{array}\right.\end{array}$$

The bistability region in the parameter space satisfies

14 $${L}_{xy}\, <\, {\gamma }_{x}{\theta }_{yx}\, <\, {U}_{xy},\quad {L}_{yx}\, <\, {\gamma }_{y}{\theta }_{xy}\, <\, {U}_{yx}.$$

There are four domains in the state space (counterclockwise from bottom right)

$$\begin{array}{rc}I:&\{x \,<\, {\theta }_{yx},y \,<\, {\theta }_{xy}\}\qquad II:\{x \,<\, {\theta }_{yx},y \,>\, {\theta }_{xy}\}\\ III:&\{x \,>\, {\theta }_{yx},y \,> \,{\theta }_{xy}\}\qquad IV:\{x \,> \,{\theta }_{yx},y\, <\, {\theta }_{xy}\}\end{array}$$

Domain II contains equilibrium FP(0,1) and IV contain equilibrium FP(1,0). We will consider domains I and III which are divided to domains of attraction of two equilibria.

#### Domain III

The equations in this domain are

15 $$\begin{array}{ll}\dot{x}&=-{\gamma }_{x}x+{L}_{xy}\\ \dot{y}&=-{\gamma }_{y}y+{L}_{yx}\end{array}$$

and the solution is

16 $$x(t)=\frac{{L}_{xy}}{{\gamma }_{x}}+\left({x}_{0}-\frac{{L}_{xy}}{{\gamma }_{x}}\right){e}^{-{\gamma }_{x}t},\quad y(t)=\frac{{L}_{yx}}{{\gamma }_{y}}+\left({y}_{0}-\frac{{L}_{yx}}{{\gamma }_{y}}\right){e}^{-{\gamma }_{y}t}.$$

Note that as *t* → *∞* this solution converges to $(\frac{{L}_{xy}}{{\gamma }_{x}},\frac{{L}_{yx}}{{\gamma }_{y}})$ which by the choice (14) of the parameter region, belongs to domain I. Therefore for each initial condition (*x*_0_, *y*_0_) in domain III we can compute time *T*_*xy*_(*x*_0_, *y*_0_) where this solution crosses *θ*_*xy*_ and the time *T*_*yx*_(*x*_0_, *y*_0_) where it crosses *θ*_*yx*_. Importantly, if *T*_*xy*_(*x*_0_, *y*_0_) < *T*_*yx*_(*x*_0_, *y*_0_) then (*x*_0_, *y*_0_) is in the domain of attraction of FP(1,0) in domain IV, and if *T*_*xy*_(*x*_0_, *y*_0_) > *T*_*yx*_(*x*_0_, *y*_0_) then (*x*_0_, *y*_0_) is in the domain of attraction of FP(0,1) in domain II. Consequently, the condition *T*_*xy*_(*x*_0_, *y*_0_) = *T*_*yx*_(*x*_0_, *y*_0_) defines the boundary between these domains of attraction.

The equations for *T*_*xy*_(*x*_0_, *y*_0_) and *T*_*yx*_(*x*_0_, *y*_0_) are

17 $${\theta }_{xy}-\frac{{L}_{yx}}{{\gamma }_{y}}=\left({y}_{0}-\frac{{L}_{yx}}{{\gamma }_{y}}\right){e}^{-{\gamma }_{y}{T}_{xy}}{\theta }_{yx}-\frac{{L}_{xy}}{{\gamma }_{x}}=\left({y}_{0}-\frac{{L}_{xy}}{{\gamma }_{x}}\right){e}^{-{\gamma }_{x}{T}_{yx}}.$$

This gives

18 $${T}_{yx}=-\frac{1}{{\gamma }_{x}}\ln \frac{{\gamma }_{x}{\theta }_{yx}-{L}_{xy}}{{\gamma }_{x}{x}_{0}-{L}_{xy}}{T}_{xy}=-\frac{1}{{\gamma }_{y}}\ln \frac{{\gamma }_{y}{\theta }_{xy}-{L}_{yx}}{{\gamma }_{y}{y}_{0}-{L}_{yx}}$$

This leads to the following equation for the separatrix between two domains of attraction in domain III:

19 $${\gamma }_{y}\ln ({\gamma }_{x}{\theta }_{yx}-{L}_{xy})-{\gamma }_{x}\ln ({\gamma }_{y}{\theta }_{xy}-{L}_{yx})={\gamma }_{y}\ln ({\gamma }_{x}{x}_{0}-{L}_{xy})-{\gamma }_{x}\ln ({\gamma }_{y}{y}_{0}-{L}_{yx}).$$

Notice that the left hand side is a constant, while the right hand side depends on the values (*x*_0_, *y*_0_).

Therefore domain of attraction within domain III of the fixed point in domain IV (**FP(1,0)**) will satisfy

20 $${\gamma }_{y}\ln ({\gamma }_{x}{\theta }_{yx}-{L}_{xy})-{\gamma }_{x}\ln ({\gamma }_{y}{\theta }_{xy}-{L}_{yx}) \,<\, {\gamma }_{y}\ln ({\gamma }_{x}{x}_{0}-{L}_{xy})-{\gamma }_{x}\ln ({\gamma }_{y}{y}_{0}-{L}_{yx}),$$

while the domain of of the fixed point in domain II will have the inequality reversed.

#### Domain I

The equations in this domain are

21 $$\begin{array}{rc}\dot{x}&=-{\gamma }_{x}x+{U}_{xy}\\ \dot{y}&=-{\gamma }_{y}y+{U}_{yx}\end{array}$$

and the solution is

22 $$x(t)=\frac{{U}_{xy}}{{\gamma }_{x}}+\left({x}_{0}-\frac{{U}_{xy}}{{\gamma }_{x}}\right){e}^{-{\gamma }_{x}t},\quad y(t)=\frac{{U}_{yx}}{{\gamma }_{y}}+\left({y}_{0}-\frac{{U}_{yx}}{{\gamma }_{y}}\right){e}^{-{\gamma }_{y}t}.$$

These equations are analogous to (22) with constants *L*_*xy*_, *L*_*yx*_ replaced by *U*_*xy*_, *U*_*yx*_, respectively. Therefore, in analogy with (19), we have the following equation for the separatrix between two domains of attraction in domain I:

23 $$\begin{array}{l}{\gamma }_{y}\ln ({\gamma }_{x}{\theta }_{yx}-{U}_{xy})-{\gamma }_{x}\ln ({\gamma }_{y}{\theta }_{xy}-{U}_{yx})={\gamma }_{y}\ln ({\gamma }_{x}{x}_{0}-{U}_{xy})\\-{\gamma }_{x}\ln ({\gamma }_{x}{y}_{0}-{U}_{yx}).\end{array}$$

We use equations (19) and (23) to classify RACIPE-selected initial conditions (*x*_0_, *y*_0_) as belonging to the basin of attraction of either of the stable equilibria in domains II and IV.

Similar equations can be written for switching system of DA, where domain I contains FP(0,0) and domain III contains FP(1,1). The corresponding equations defining the boundaries of the basins of attraction are:

24 $${\gamma }_{y}\ln ({\gamma }_{x}{\theta }_{yx}-{L}_{xy})-{\gamma }_{x}\ln ({\gamma }_{y}{\theta }_{xy}-{U}_{yx})={\gamma }_{y}\ln ({\gamma }_{x}{x}_{0}-{L}_{xy})-{\gamma }_{x}\ln ({\gamma }_{x}{y}_{0}-{U}_{yx}).$$

for domain I and

25 $${\gamma }_{y}\ln ({\gamma }_{x}{\theta }_{yx}-{U}_{xy})-{\gamma }_{x}\ln ({\gamma }_{y}{\theta }_{xy}-{L}_{yx})={\gamma }_{y}\ln ({\gamma }_{x}{x}_{0}-{U}_{xy})-{\gamma }_{x}\ln ({\gamma }_{x}{y}_{0}-{L}_{yx}).$$

for domain III respectively.

### Translating Shifted Hill functions to DSGRN

The RACIPE model for the dynamics of a node *k* is given by

26 $${\dot{x}}_{k}={P}_{k}\mathop{\prod}\limits_{j\to k}\frac{{H}^{s}({x}_{j},{\theta }_{kj},{n}_{kj},{a}_{kj})}{{a}_{kj}}\mathop{\prod}\limits_{j \dashv k}{H}^{s}({x}_{j},{\theta }_{kj},{n}_{kj},{i}_{kj})-{\gamma }_{k}{x}_{k}.$$

with the requirement that *a* > 1 and *i* < 1. Shifted Hill functions are monotone sigmoid functions. In particular, as *n* → *∞*, they converge to switching functions. We note that *H*^*s*^(0) = 1 and

$$\mathop{\lim }\limits_{x\to \infty }{H}^{s}=a\,{{\mbox{or}}}\,i.$$

It follows that the range of *H*^*s*^ for activating edges is (1, *a*), and for inhibiting edges is (*i*, 1). Since the effect of an activating edge in (26) is ${\widetilde{H}}^{s}:= \frac{{H}^{s}}{a}$, this range of ${\widetilde{H}}^{s}$ is $(\frac{1}{a},1)$. The final parameter of RACIPE formalism is the basal production rate *P*_*k*_ at each network node.

The DSGRN model for the dynamics of node *k*, assuming product interactions between edges with common target node, is given by

27 $${\dot{x}}_{k}=\mathop{\prod}\limits_{j\to k}{\sigma }^{+}({x}_{j},{\theta }_{kj},{L}_{kj},{U}_{kj})\mathop{\prod}\limits_{j \dashv k}{\sigma }^{-}({x}_{j},{\theta }_{kj},{L}_{kj},{U}_{kj})-{\gamma }_{k}{x}_{k}.$$

The switching functions *σ*^+^ and *σ*^−^ are defined in (7). We first observe that in the limit *n* → *∞* a half-saturation parameter *θ*_*kj*_ in *H*^±^ of (26) becomes the threshold *θ*_*kj*_ in functions *σ*^±^. Since they are equivalent, we have chosen to use the same notation in both formalisms.

We now address conversion between the fold multiplication parameters *a*_*kj*_, *i*_*kj*_ and basal rate *P*_*k*_ in (26) and parameters *L*_*kj*_ and *U*_*kj*_ in (27). At first glance, there are more parameters in the DSGRN model than in the RACIPE formulations. When the inputs to a node *k* combine as a product, which is the case in both (26) and (27), the key observation is that the highest value of this product at node *k* is *P*_*k*_ in (26) and ∏_*j*→*k*_*U*_*kj*_ in (27). Therefore a natural correspondence is

28 $${P}_{k}=\mathop{\prod}\limits_{j\to k}{U}_{kj}.$$

#### Converting a RACIPE Model to a DSGRN Model

Given a RACIPE model, we choose to evenly attribute the basal production rate, *P*_*k*_, to each input edge by setting

29 $${U}_{kj}:= {P}_{k}^{\frac{1}{{m}_{k}}},$$

where *m*_*k*_ is the number of inputs to node *k*. The corresponding DSGRN parameters are given by

30 $$\,{{\mbox{for}}}\,j\to k\qquad {L}_{kj}:= {P}_{k}^{\frac{1}{{m}_{k}}}\frac{1}{{a}_{kj}}$$

31 $$\,{{\mbox{for}}}\,j \dashv k\qquad {L}_{kj}:= {P}_{k}^{\frac{1}{{m}_{k}}}{i}_{kj}$$

With this choice of correspondence in parameters each shifted Hill function in the RACIPE model converges to the corresponding switching function in the DSGRN model as *n* → *∞*. A consequence of Theorem 3.11 of^18^ is that the fixed points of the DSGRN model are in one-to-one correspondence to the fixed points of the RACIPE model for large enough *n*.

#### Converting a DSGRN Model to a RACIPE Model

Given DSGRN model, we first compute the basal rate *P*_*k*_ for each *k* from (28). Let *m*_*k*_ be the number of incoming edges to node *k*. Then the conversion between *L*_*kj*_ and *a*_*kj*_, *i*_*kj*_ is as follows:

32 $$\,{{\mbox{for}}}\,j\to k\qquad {a}_{kj}:= \frac{{P}_{k}^{\frac{1}{{m}_{k}}}}{{L}_{kj}}$$

33 $$\,{{\mbox{for}}}\,j \dashv k\qquad {i}_{kj}:= \frac{{L}_{kj}}{{P}_{k}^{1{m}_{k}}}$$

In addition, since DSGRN corresponds to a limit as Hill coefficients approach infinity, the Hill coefficient of RACIPE is *n*_*kj*_ = *∞* for each *k*, *j*.

We remark that in this paper, we only use the conversion from RACIPE to DSGRN. While the reverse operation is defined, it has some undesirable properties. Let *R* be the transformation from a RACIPE parameter sample to a DSGRN parameter sample and let *D* be the transformation from a DSGRN parameter sample to a RACIPE parameter sample. Then it is easy to check that *D* ∘ *R* = *Id*. On the other hand, note that *R* ∘ *D* ≠ *Id*. In fact, some valid DSGRN parameter samples do not result in valid RACIPE parameters, because the assignment produces a RACIPE parameter with *a*_*kj*_ = 1 or *i*_*kj*_ = 1. Therefore, *D* is not well-defined over all of parameter space in the DSGRN framework.

A more subtle problem is the following. Let $\bar{{{{\mathcal{P}}}}}\subset {{{\mathcal{P}}}}$ be the domain on which *D* is well-defined, and let $p\in \bar{{{{\mathcal{P}}}}}$. Then one can check that *q* = *R* ∘ *D*(*p*) ≠ *p*, and, furthermore, *p*, *q* may not be associated to same parameter node in the DSGRN parameter graph. Therefore, we advise caution when interpreting the meaning of RACIPE parameter samples that emerge from sampling in DSGRN parameter space followed by transformation under *D*.

### Reporting summary

Further information on research design is available in the Nature Research Reporting Summary linked to this article.

## Supplementary information

SUpplementary figures

Reporting Summary

## Data Availability

All raw and processed data files are available at the link: https://github.com/Cancer-Systems-Biology-Lab/RACIPEvDSGRN.

## Code availability

All the codes used for analysis are available at the link: https://github.com/Cancer-Systems-Biology-Lab/RACIPEvDSGRN.
