# Supplementary material for: Assessing biological network dynamics: comparing numerical simulations with analytical decomposition of parameter space
Source: NPJ Syst Biol Appl. 2023 Jul 3;9:29. doi: 10.1038/s41540-023-00289-2 (PMC10318016; doi:10.1038/s41540-023-00289-2)
Supplement: Supplementary file 1 — SUpplementary figures [file 41540_2023_289_MOESM1_ESM.pdf]

# 1 Supplementary Figures

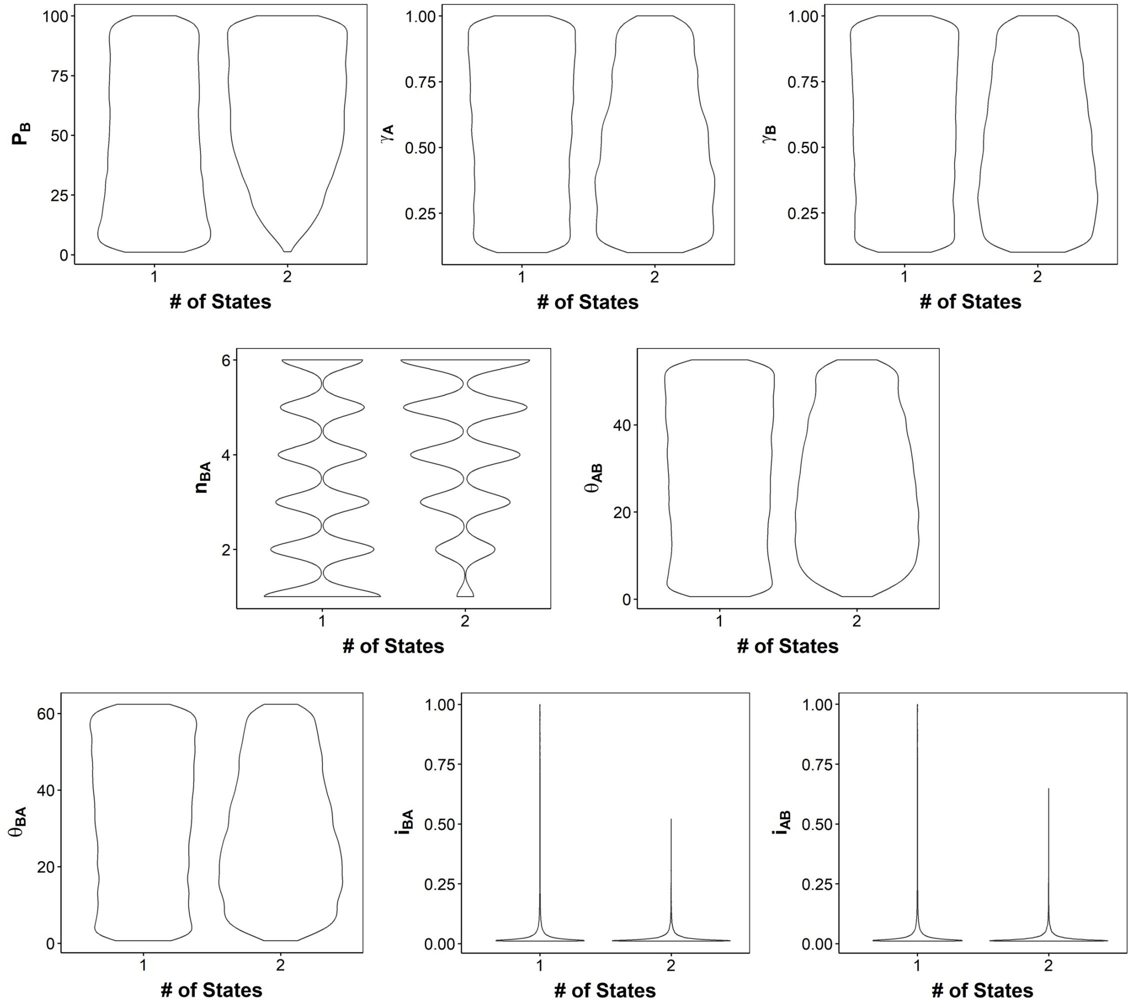


**Supplementary Figure 1 :** Parameter distribution for monostable and bistable parameter sets for TS in RACIPE

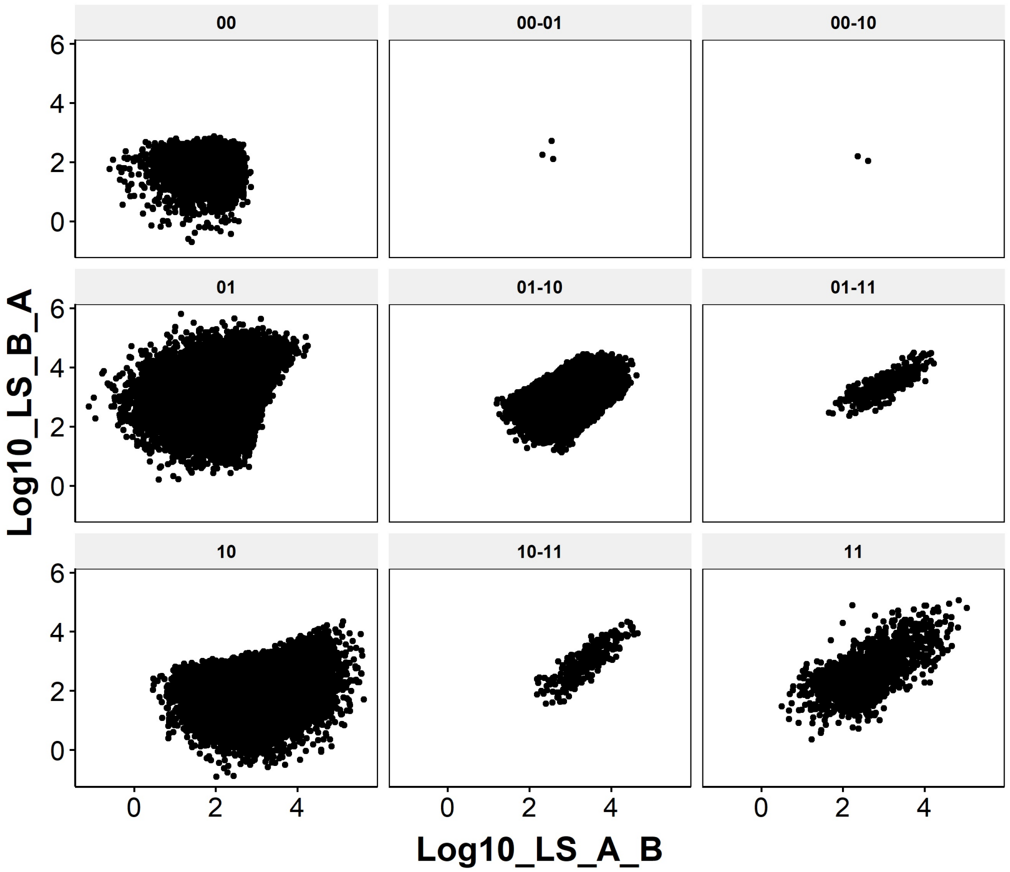


**Supplementary Figure 2 :** Scatterplot showing the Link strength values for parameters corresponding to different attractor-repertoires.

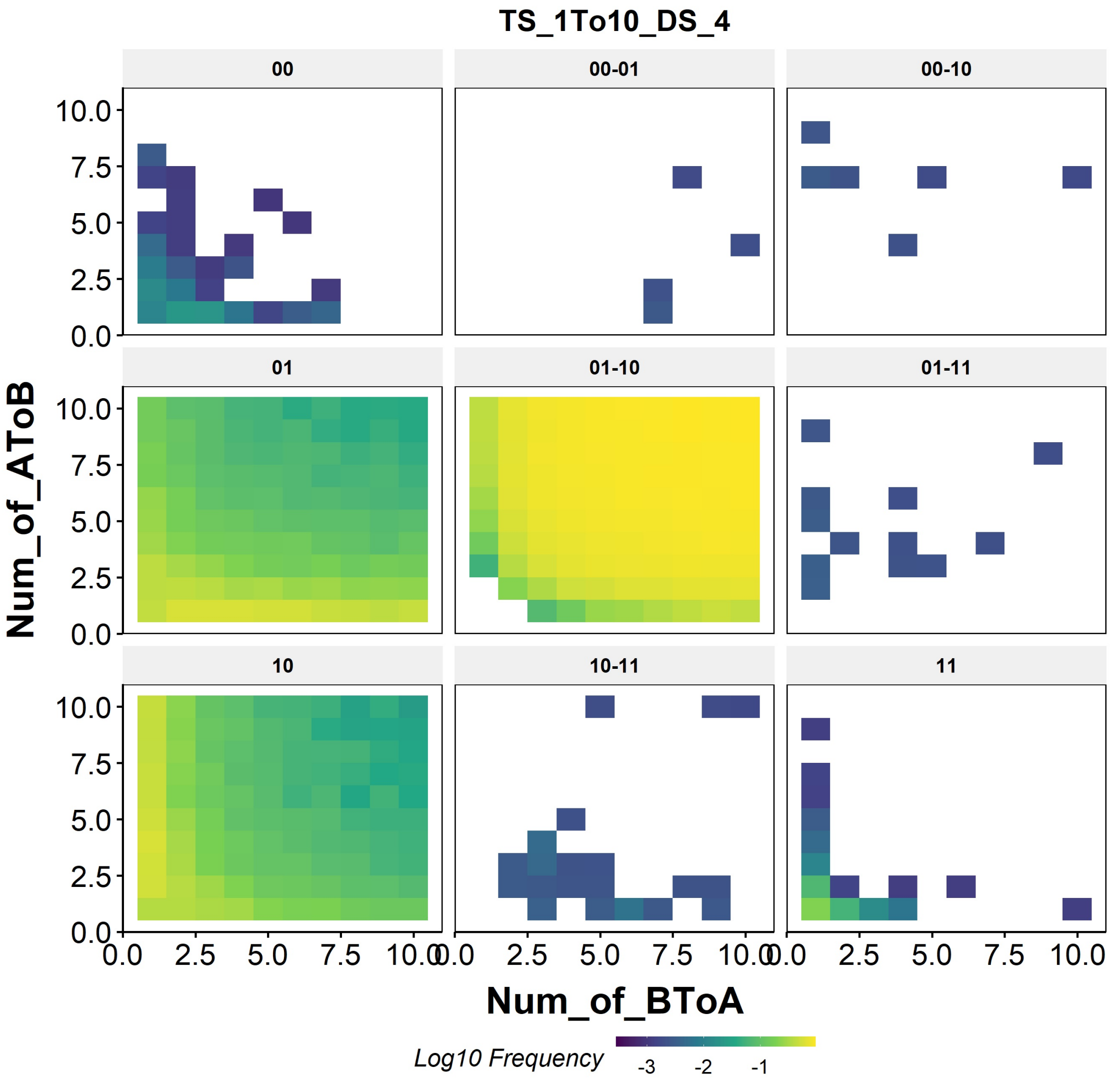


**Supplementary Figure 3 :** Dependence of attractor-repertoire frequency of TS in parameter node 4 (bistable parameter node) on Hill coefficients in the 1-10 range. The color denotes, in log scale, the frequency of a attractor-repertoire for a given combination of Hill coefficients.

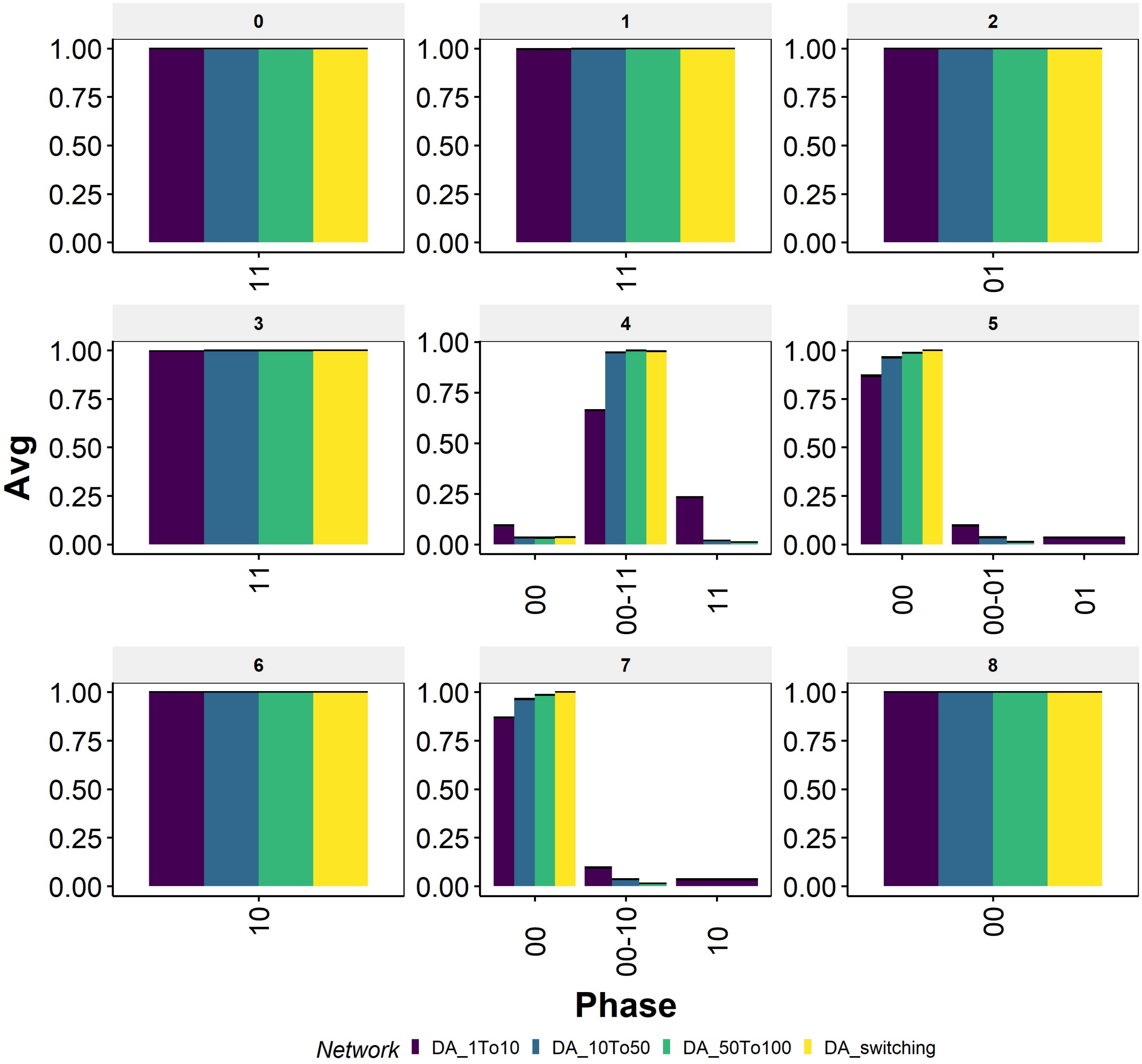


**Supplementary Figure 4 :** Attractor-repertoire distribution of switching system against different ranges of Hill coefficients in RACIPE for DA. The switching system is represented by yellow colored bars. Default RACIPE conditions are represented by the dark blue colored bars. The range of Hill coefficients in each case is reported in the color-legend.

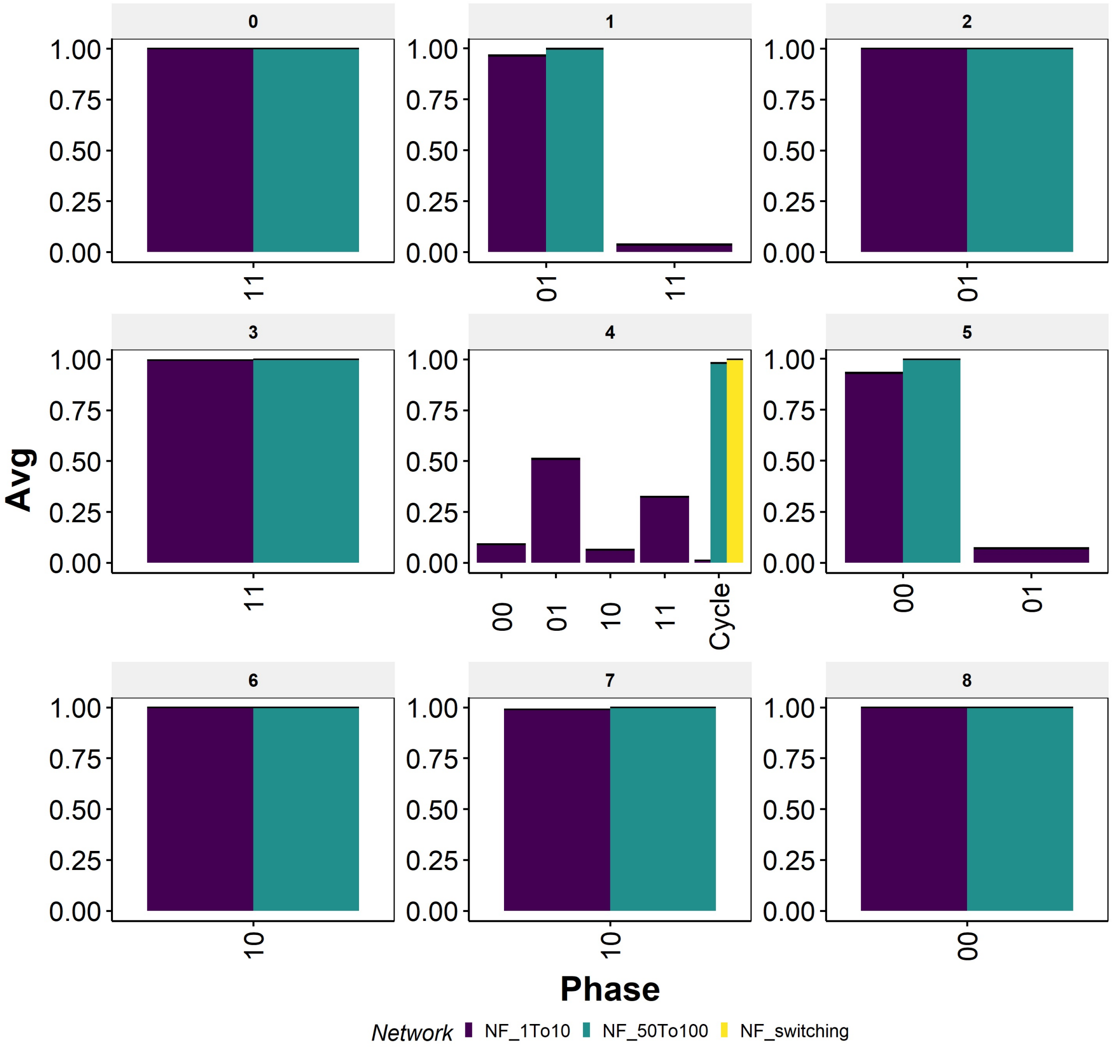


**Supplementary Figure 5 :** Attractor-repertoire distribution of switching system against different ranges of Hill coefficients in RACIPE for NF. The switching system is represented by yellow colored bars. Default RACIPE conditions are represented by the dark blue colored bars. The range of Hill coefficients in each case is reported in the color-legend.

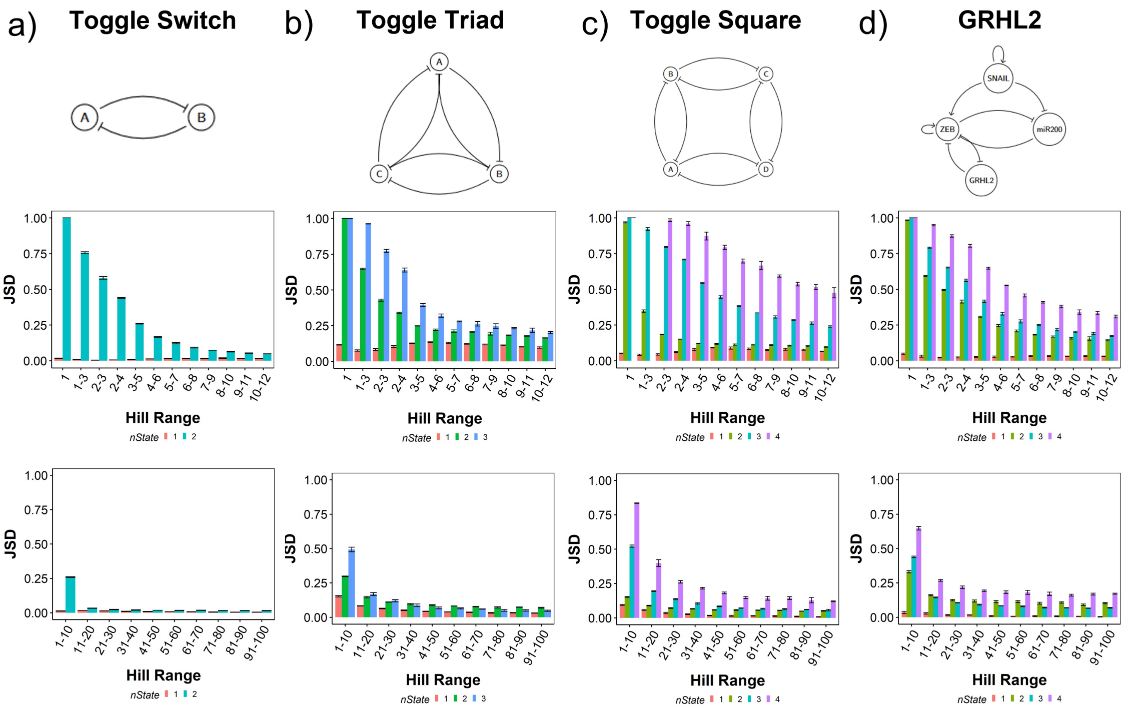


**Supplementary Figure 6 :** JSD between the frequency distribution of the attractor repertoire obtained from DSGRN and RACIPE for a) TS and b) TT.

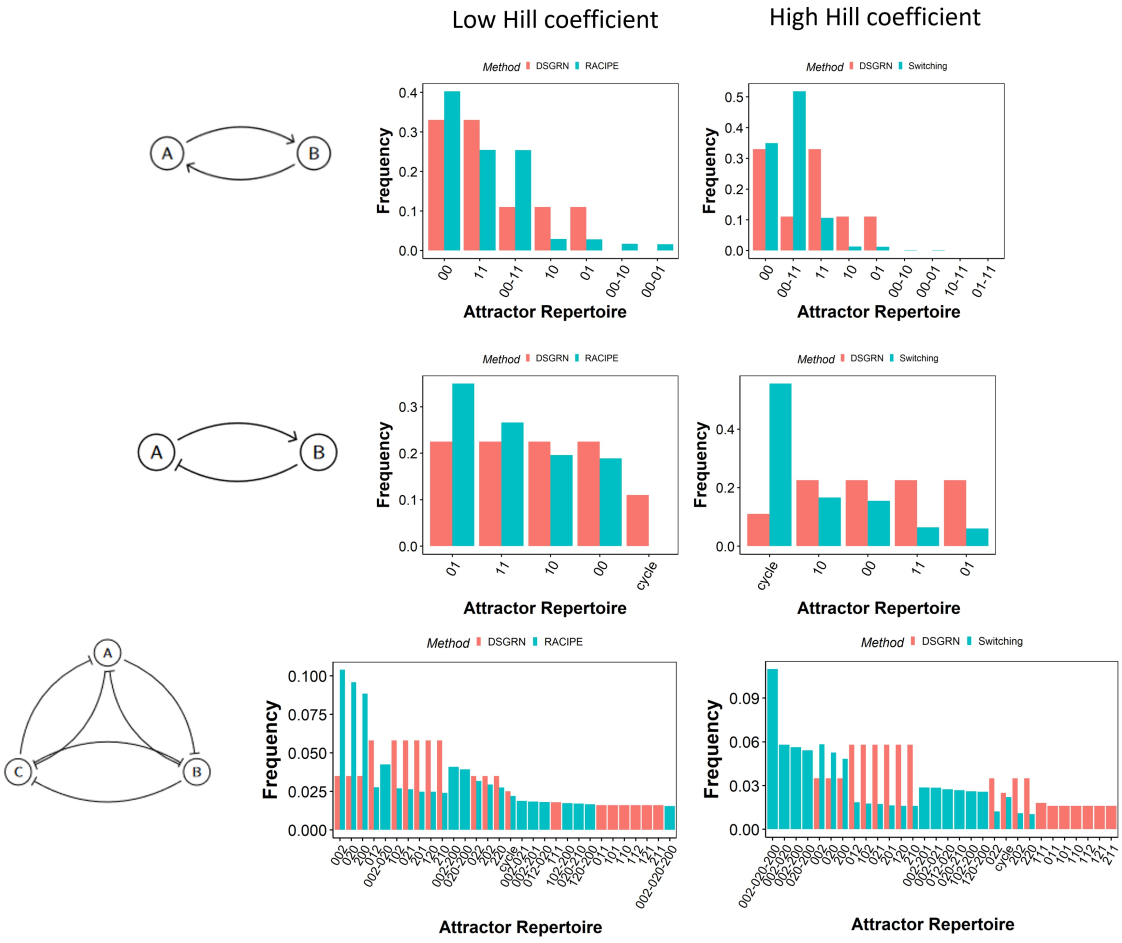


**Supplementary Figure 7 :** Comparison of the DSGRN attractor-repertoire distribution with that of RACIPE at low and high Hill coefficients for DA, NF and TT.
